# Nitrogen vacancy defects in single-particle nanodiamonds sense paramagnetic transition metal spin noise from nanoparticles on a transmission electron microscopy grid†

**DOI:** 10.1039/d3na00155e

**Published:** 2023-08-18

**Authors:** Bradley T. Flinn, Valentin Radu, Michael W. Fay, Ashley J. Tyler, Jem Pitcairn, Matthew J. Cliffe, Benjamin L. Weare, Craig T. Stoppiello, Melissa L. Mather, Andrei N. Khlobystov

**Affiliations:** a School of Chemistry, University of Nottingham, University Park Nottingham NG7 2RD UK andrei.khlobystov@nottingham.ac.uk; b Optics and Photonics Group, Faculty of Engineering, University of Nottingham, University Park Nottingham NG7 2RD UK melissa.mather@nottingham.ac.uk; c Nanoscale and Microscale Research Centre, University of Nottingham Nottingham NG7 1QL UK; d Centre for Microscopy and Microanalysis, University of Queensland. St Lucia 4072 Australia

## Abstract

Spin-active nanomaterials play a vital role in current and upcoming quantum technologies, such as spintronics, data storage and computing. To advance the design and application of these materials, methods to link size, shape, structure, and chemical composition with functional magnetic properties at the nanoscale level are needed. In this work, we combine the power of two local probes, namely, Nitrogen Vacancy (NV) spin-active defects in diamond and an electron beam, within experimental platforms used in electron microscopy. Negatively charged NVs within fluorescent nanodiamond (FND) particles are used to sense the local paramagnetic environment of Rb_0.5_Co_1.3_[Fe(CN)_6_]·3.7H_2_O nanoparticles (NPs), a Prussian blue analogue (PBA), as a function of FND-PBA distance (order of 10 nm) and local PBA concentration. We demonstrate perturbation of NV spins by proximal electron spins of transition metals within NPs, as detected by changes in the photoluminescence (PL) of NVs. Workflows are reported and demonstrated that employ a Transmission Electron Microscope (TEM) finder grid to spatially correlate functional and structural features of the same unique NP studied using NV sensing, based on a combination of Optically Detected Magnetic Resonance (ODMR) and Magnetic Modulation (MM) of NV PL, within TEM imaging modalities. Significantly, spin–spin dipole interactions were detected between NVs in a single FND and paramagnetic metal centre spin fluctuations in NPs through a carbon film barrier of 13 nm thickness, evidenced by TEM tilt series imaging and Electron Energy-Loss Spectroscopy (EELS), opening new avenues to sense magnetic materials encapsulated in or between thin-layered nanostructures. The measurement strategies reported herein provide a pathway towards solid-state quantitative NV sensing with atomic-scale theoretical spatial resolution, critical to the development of quantum technologies, such as memory storage and molecular switching nanodevices.

## Abstract 2

Non-integrated correlative light-electron microscopy with nitrogen vacancy sensing on transmission electron microscopy finder grids for the study of paramagnetic Prussian blue analogue nanoparticles.

## Graphical abstract

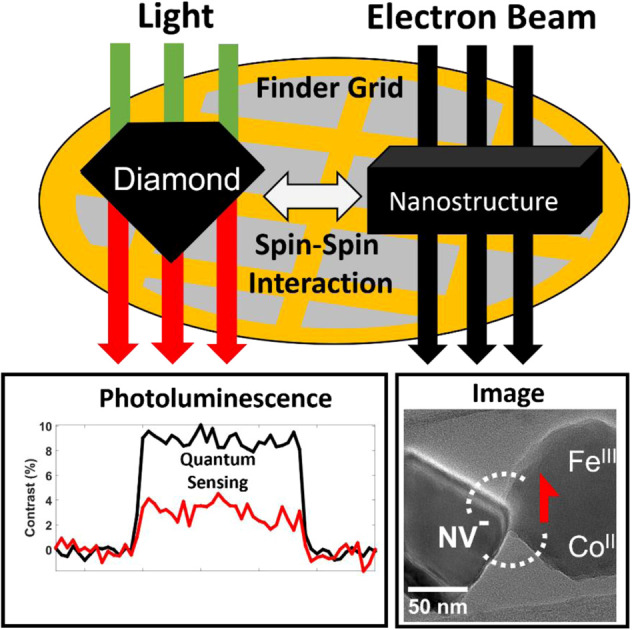

## Introduction

There is a universal and ongoing need for the development of new methods capable of pushing the boundaries of measurement to sustain frontier science. Advances in materials science and therapeutics are driving the establishment of new measurement tools to map and sense spin-active particles and molecules down to the nanoscale level. The realisation of this is challenging and requires advanced techniques including electron microscopy,^1,2^ scanning probe techniques^1,2^ and superconducting quantum interference device (SQUID) microscopy.^3^ To be effective, measurement techniques must provide high spatial resolution, high sensitivity and be relatively non-invasive. One candidate scanning probe technique is magnetic force microscopy (MFM), which uses a magnetically coated atomic force microscopy (AFM) tip to probe local magnetic forces and mechanical properties at the nanoscale.^4^ Spatial resolution of MFM is generally limited to the sharpness of the tip, giving a variety of different reported resolutions (approximately 10 nm).^5^ MFM provides contour maps, mainly targeted for ferromagnetic materials and is viewed often as only a qualitative technique due to challenges of resolving the magnetic field from other artefacts acting on the measurement, such as chemical forces, topology and the tip itself being magnetic and invasive.^6^

**Fig. 1:**
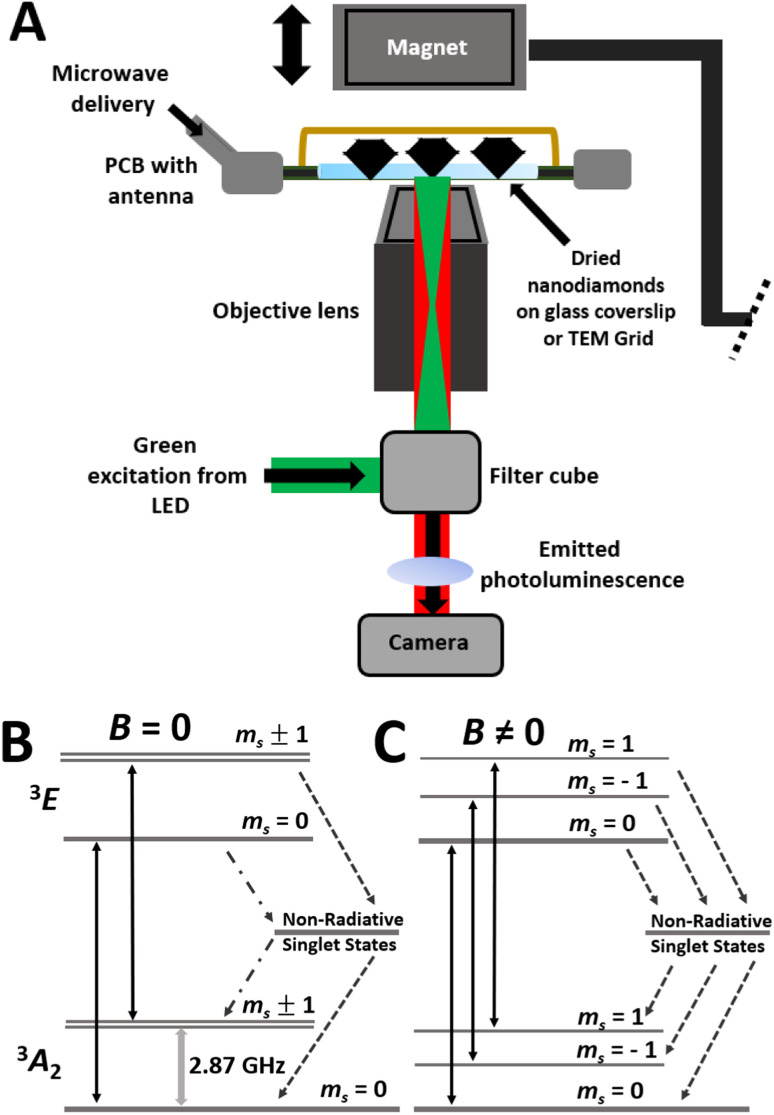
(A) A scheme of experimental set-up showing FNDs on a glass coverslip or TEM grid within a custom-made PCB with a wire antenna for microwave delivery. A magnet is also shown which is placed directly above the dried FNDs for the delivery of a magnetic field. Light illumination and detection pathways have also been shown. Schematic Jablonski diagrams showing the excitation and decay pathways of the NV^−^ centre without (B) and with an off axis magnetic field (C). Transitions between ground and excited triplet states (solid arrows) as well as microwave stimulation are shown with non-radiative singlet state pathways, weak (-•-) and strong (--), also highlighted. The off axis magnetic field defines the quantization axis, causes spin mixing in the ground and excited states, and leads to all non-radiative transition rates being likely to be non-zero.

Nitrogen Vacancy (NV) centres in diamond are emerging as leading contenders in the field of nanoscale sensing, owing to the intrinsic spin interaction of NVs to magnetic fields and external spins.^7^ Advantageously, NVs are photostable quantum probes whose spin state can be addressed and read out optically using visible light. Perturbation of the NV photophysics by changes in the magnetic environment local to the NV enables magnetic field measurements at the nanoscale.^8^ This naturally occurring paramagnetic impurity comprises a substitutional nitrogen atom adjacent to a vacant site in the diamond lattice, which in the negative charge state (NV^−^) forms a spin triplet, *m*_s_ = −1, 0, and +1 (Fig. 1B and C).^8,9^ Upon excitation with green light, the NV^−^ centre produces broadband fluorescent emission extending into the near infrared region with a zero-phonon line at 637 nm.^8^ More recently, the NV^−^ centre has attracted attention as a potential fluorescent probe for use in quantum technological applications due to its high quantum yield and robust luminescence, which does not bleach.^10,11^ One exciting application is the use of NV^−^ centres in diamond for data storage materials, utilising light to read and write data sets.^12^ The ability to optically manipulate and monitor the electron spin state in the NV^−^ centre is core to its quantum sensing capabilities. NV^−^ is the active charge state for electron spin related applications in physics, such as ambient temperature optically detected magnetic resonance (ODMR).^13–17^ Having the ability to detect spin-active nanostructures *via* optical readout down to the single spin level, with high spatial resolution, could have a wide range of quantum computing applications.^18^ Recently, the spatial resolution of magnetically sensitive scanning probe techniques has been improved through the development of specialist probes incorporating NV centres, which can be scanned across a substrate and addressed optically.^19^ Scanning NV magnetometry can be performed at ambient temperature and pressure opening up more possibilities for paramagnetic material sensing. However, this technique has complex instrumentation design, requires careful control of probe to sample standoff distance, with relatively long acquisition times intrinsic to scanning based imaging methods.

The work herein addresses the need for nanoscale functional and structural mapping of spin-active nanomaterials by combining NV^−^ sensing data obtained on a light microscope with TEM imaging techniques. At the core of this work is the use of TEM finder grids, functionalised with fluorescent nanodiamonds (FNDs), as the experimental platform for sample loading and analysis. This facilitates workflows for spatial correlation of images obtained from light and electron microscopy providing new experimental protocols to correlate nanoscale structure and chemical composition with magnetic, oxidation and electronic states of matter down to the single-particle level.

The experimental strategy reported here extends the reach of correlative light-electron microscopy (CLEM), that combines the throughput and versatility of the light microscope with the higher magnification and resolving power of the electron microscope, as well as the ability to probe local chemical composition by EM. Previously CLEM has been successfully applied to tracking nanosized diamonds in cells,^20–22^ even imaging and quantifying NDs down to the single particle level.^23^ To our knowledge, the full potential of CLEM combined with NV^−^ sensing for detecting local external magnetic environments (using TEM methods to precisely image and measure distance in 3D on corresponding area/interacting particles) has not been realised. Related work demonstrate the concept of this using non-integrated CLEM, where light and electron microscopy images are obtain on different instruments, with NV^−^ sensing on TEM grids, to studying magnetism and phase transitions down to the single particle level.^24,25^ Herein we demonstrate advancement in this non-integrated CLEM NV^−^ sensing methodology for identical-location (IL) spin-sensing analysis of paramagnetic Prussian blue analogue (PBA) nanoparticles (NPs), Rb_0.5_Co_1.3_[Fe(CN)_6_]·3.7H_2_O = PBA throughout, down to the single-particle level. Workflows for acquisition of accurate functional information, while providing spatial resolution down to the atomic level, are detailed using commercially available electron microscopy grids (Lab-On-A-Grid methodology) which can be stored and transferred reliably between different instruments with ease. We report perturbation of NV^−^ spins by proximal spin fluctuations in paramagnetic metal centres in PBA NPs as a function of FND-PBA separation and local PBA concentration (number density of NPs), as determined by changes in FND PL. Using TEM tilt series and electron energy-loss spectroscopy (EELS) analysis we determine NP interactions, in 3-dimensional (3D) space, on a TEM grid. Significantly, nanoscale quantification of FNDs and PBA spatial separation is performed enabling the distance dependence of through space spin–spin interactions between NV centres and spin active centres in PBA to be studied *via* changes in NV^−^ contrast. Correspondingly this enabled the sensing range of the FNDs, for detection of spin-active centres in PBA, to be evaluated and the rapid reduction in sensitivity with increase in FND to PBA separation to be studied. It is noted that for individual NVs, dipole–dipole interaction strength varies as an inverse cube of distance.^24,25^ Our work advances IL-TEM and light microscopy imaging for sensing magnetic nanomaterials, thus opening potential for applications for spintronic, data storage and switchable magnetic nanodevices.^26–28^

## Experimental

### Materials

#### Fluorescent nanodiamond samples

Fluorescent nanodiamonds (FNDs throughout) were purchased from FND Biotech Inc (brFND-100). Average FND diameter 100 nm containing >1000 NV centres per FND particle.^29^ Prior to NV sensing, FNDs were either dried onto a glass coverslip (GCS) from a 0.1–0.5 mg mL^−1^ suspension and then incubated at 60 °C for at least 12 h or drop cast onto TEM grids, again using a 0.1–0.5 mg mL^−1^ FND suspension (grid left to dry in air for at least 4 hours), for identical-location light-electron microscopy measurements.

#### Rubidium cobalt iron prussian blue analogue (PBA throughout)

**Fig. 2:**
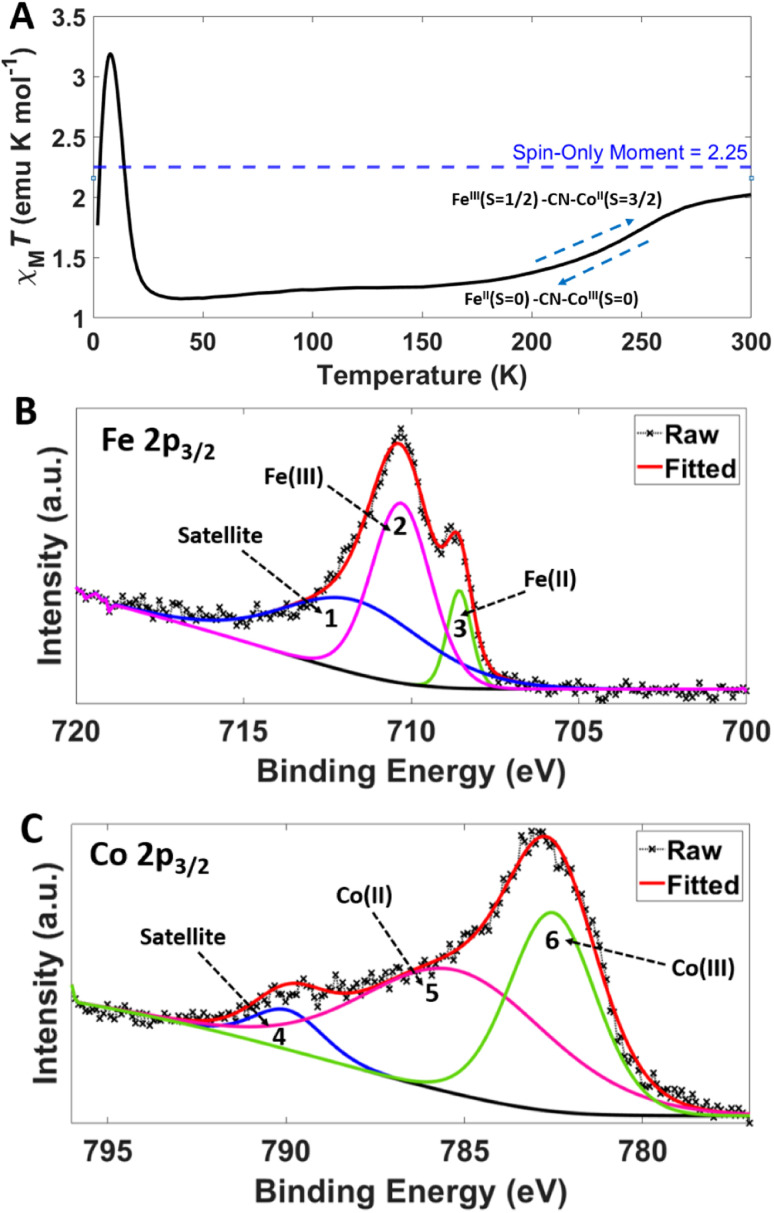
Thermal variation of the value *χ*_m_*T* for PBA (A). The blue dotted line shows as temperature increases *χ*_m_*T* approaches the high temperature spin-only limit of 2.25 emu K mol^−1^ for a non-interacting *S* = 3/2 and *S* = 1/2 pair, consistent with Co(ii)-HS and Fe(iii)-LS states. Curie constant 2.14 K emu mol^−1^ and Weiss constant −6.39 K confirming the presence of antiferromagnetic interactions at temperatures below the Curie point (14.5 K). Isothermal magnetisation M(H) measured at 2 K is shown in the ESI (Fig. S3).† High resolution XPS spectra of PBA in the Fe 2p_3/2_ (B) and Co 2p_3/2_ (C) regions. The raw and fitted data is shown along with the peaks for oxidation state and satellite evaluation (1–6). Peaks 1, 2 and 3 correspond to a satellite, Fe(iii) and Fe(ii) respectively. Peaks 4, 5 and 6 correspond to a satellite, Co(ii) and Co(iii) respectively.

PBA NPs were synthesised *via* a method adapted from Bleuzen *et al.*^30^ Prior to addition, solutions A, B and C were adjusted to pH 5. 10 mL aqueous solution containing RbNO_3_ (18.4 mg, 0.125 mmol) – solution A – was added to a 10 mL aqueous suspension containing K_3_[Fe(CN)_6_] (65.8 mg, 0.2 mmol) – solution B. To this, was dropwise added an 80 mL aqueous solution containing Co(NO_3_)_2_·6H_2_O (58.2 mg, 0.2 mmol) – solution C. The addition rate was regulated to last 3 hours at room temperature with stirring. Immediately upon addition a dark purple dispersion formed. When the addition was complete, the reaction mixture was stirred for a further 30 min before an equal volume of deionised water was added to the dispersion and centrifuged at 8000 rpm for 15 min (3 repeats). The final solutions after the third centrifugation step were combined, vacuum filtered and dried in air, affording a dark purple powder, yield 32.1%; elemental microanalysis found: C 17.35, H 2.03, N 19.73% w/w (calculated: C 18.11, H 1.85, N 21.13% w/w for Rb_0.5_Co_1.3_[Fe(CN)_6_]·3.7H_2_O. Concentration of paramagnetic centres (Fe and Co), assuming entirely the high temperature phase (Fig. 2A), is ∼90 000 ppm). IR-ATR (*υ* max, cm^−1^): 2116 (C≡N), 595 (Fe^III^–CN), 530 (Co^II^–CN).

### Methods

#### Nitrogen-vacancy paramagnetic sensing

The NV^−^ based sensing protocol involved detection of PL from NV^−^ centres in FND particles by sweeping the microwave (MW) frequency, commonly known as ODMR, or varying the strength of an externally applied off axis magnetic field – in magnetic modulation (MM) measurements.^31–34^ A signal generator (Agilent E4428C ESG) and MW amplifier (AR 20S1G4 MW amplifier, gain 0 dBm, power 30%) were used to deliver MWs *via* a coated 0.125 mm diameter straight copper wire electrically connected to 50 Ohm tracks on a custom-designed printed circuit board (PCB). For ODMR studies the microwave frequency was swept from 2.77 GHz to 2.97 GHz in 2 MHz steps to probe the ground state NV^−^ spin transitions. In MM, the off axis magnetic field was applied *via* an electromagnet placed in close proximity to the sample. A signal generator (Tektronix AFG 3102) delivered a square wave that cycled between 0 V and 10 V at a frequency of 500 mHz (coverslips) or 50 mHz (grids) to modulate the applied magnetic field strength between 0 mT and 40 mT, measured by a transverse Hall probe located on the microscopy stage.

The experimental setup (Fig. 1A) consisted of a GCS with adsorbed FNDs (TEM finder grid studies consisted of FNDs dried onto grids which were placed onto a clean GCS) mounted on the custom-designed PCB, designed to have an open aperture to enable illumination of the sample *via* an inverted fluorescence microscope (Olympus IX83). NV centres were illuminated with a CoolLED Pe-4000 Illumination System (LED) filtered through a bandpass excitation filter centred at 545 nm (ex. 530–560 nm). The light was subsequently focused on the back focal plane of an oil-immersion 60× objective lens (NA = 1.42) providing a power density of 5.08 mW cm^−2^ at the sample position. Emitted PL was filtered through a 575 nm long-pass filter and imaged onto a scientific complementary metal-oxide-semiconductor (sCMOS) camera (Photometrics 95 Prime B) providing a maximum field of view of 200 μm × 200 μm. The temperature at the sample plane, measured using a non-contact infrared probe (Amprobe IR-710 Infrared Thermometer), did not exceed 28 °C.

The detection of the paramagnetic environment was first carried out by ODMR spectra using a continuous wave (CW) measurement regime in which incoherent light from the LED constantly illuminated the sample whilst images of PL were acquired as the MW frequency was swept to probe the ground state NV^−^ spin transitions. Image acquisition was synchronized with MW sweeps using an Olympus Real-Time Controller (RTC) *via* the Olympus CellSens software (Tokyo, Japan). The resulting images at each frequency were analysed to extract the PL intensity, determined by averaging pixel intensities from regions of interest (ROI), over which the illumination (LED) intensity was uniform. At each frequency, 10 repeats of measurements were performed. ODMR spectra were produced by plotting average PL intensity from all repeats against MW frequency. ODMR spectra were then normalized by dividing all average PL intensities by the maximum averaged intensity within the sweep, giving normalised PL in arbitrary units (a.u). For MM measurements, samples were constantly illuminated, and images of PL acquired as the external magnetic field strength was varied. For each image frame ROIs were chosen and average PL intensity calculated. MM was visualised by plotting average PL intensity against image frame number (image frame exposure time: 30 ms for GCS, 307 ms for finder grids). PL intensity was then normalised with respect to the baseline values obtained under the external magnetic field, giving contrast in % − contrast (%) = [(Raw PL)/(MagnetOn PL) − 1] × 100. The difference in PL (%) under the external magnetic field did not exceed 25–30%, quoted for a single NV with a perfectly aligned magnetic field.^35^ For all measurements the optically induced NV polarization rate was in a regime where the polarization rate is proportional to the excitation power and where intrinsic and external spin lattice relaxation processes contribute to changes in PL contrast.^36^ For both ODMR and MM, proximal spin-active species decreased PL contrast (contrast reduction throughout). Quantitative MM values are extracted from ‘magnet off’ states, quoted in percentage change in PL upon PBA addition. There is assumed to be no residual magnetisation from ‘magnet on’ frames, therefore PBA NPs are assumed to be in a paramagnetic spin state during analysis ‘off’ frames.

#### Scanning and transmission electron microscopy

(S)TEM images were collected on JEOL 2100+ and JEOL 2100F FEG TEM instruments using a 200 kV accelerating voltage (resolution limit 0.19 nm). Bright field-TEM images were collected on OriusSC1000, Ultrascan1000XP and OneView Gatan cameras. STEM Images were collected using a JEOL Dark Field (DF)-STEM detector. Tomography tilts were completed around the *x*-axis using a Gatan 916 room temperature tomography holder from −50 to +50°. Image analysis was performed on the ImageJ software to give *d*-spacing, particle size and distance information.^37^

#### Electron paramagnetic resonance (EPR) spectroscopy

EPR spectra were recorded on a Bruker EMX spectrometer using Quartz glass tubes at room temperature in the X-band.

#### Infrared spectroscopy

Attenuated total reflectance spectra were taken using a Bruker ALPHA FT-IR instrument. Samples were analysed in the purely solid state.

#### Energy dispersive X-ray spectroscopy

EDX spectra were acquired for samples mounted on lacey carbon copper TEM finder grids using an X-Max 100TLE, AZTEC software was used for data analysis.

#### X-ray photoelectron spectroscopy

XPS was performed using a Kratos AXIS SUPRA PLUS instrument with a monochromatic Al Kα X-ray source (*hν* = 1486.6 eV) operated at room temperature with 10 mA emission current and 12 kV anode potential. The electron collection spot size was *ca.* 700 × 300 μm^2^. A pass energy of 160 eV was used for the survey scans and 20 eV for the high-resolution scans. Spectra were converted into VAMAS format for further analysis.

#### Electron energy-loss spectroscopy

EELS spectra were taken on a Gatan Enfinium SE spectrometer. Accurate background subtraction was performed using a previously published MATLAB script^38^ which was adapted to obtain thickness measurements using the log-ratio technique.^39^ A full MATLAB script can be found in the additional files. Zero-loss peaks (ZLP) were fitted between the full width half maxima (FWHM) and where the peak meets the baseline. Plasmon end values were also chosen when the peak plateaued into the baseline. Details of EELS analysis for thickness measurements, with an example, can be found in the ESI, Fig. S20.†

#### Dynamic light scattering

DLS size particle data and zeta potential measurements were taken on a Zetasizer Nano ZS instrument (zeta potential measurements using disposable folded capillary cells). All measurements were taken in water (pH ∼ 7) at room temperature.

#### Powder X-ray diffraction

PXRD measurements were performed using a PANalytical X'Pert Prodiffractometer equipped with a Cu-Kα radiation source (*λ* = 1.5432 Å, 40 kV, 40 mA) in Bragg–Brentano geometry using a Si zero background holder. All samples were wetted with acetone to aid sample adhesion.

#### Magnetic property measurements

Magnetisation measurements were carried out using a Quantum Design Magnetic Property Measurement System (MPMS XL) superconducting quantum interference device (SQUID) magnetometer in the temperature range 2 K to 300 K at a field of 1 T. Diamagnetic corrections were made using Pascal's constants. Magnetisation *vs.* field measurements were also performed at 2 K using a field range of −5 T to 5 T.

#### Correlative light-electron microscopy using TEM finder grids

An aqueous 0.1–0.5 mg mL^−1^ FND solution (1 mL) was sonicated (10 min) and added dropwise onto a suspended copper 200 mesh lacey carbon finder grid (Agar) and allowed to dry directly in air (>4 hours). Fluorescence microscopy was then used to survey grid locations which had well dispersed FND particles. At this stage ODMR and MM measurements were taken of the FND dispersed on the grid. The finder grid was next transferred to TEM to image FND particles at a low electron beam (e-beam) flux (50 e nm^−2^ s^−1^ to 1000 e nm^−2^ s^−1^) in specific locations. ODMR and MM measurements were now repeated to ascertain the effect of the e-beam on NV^−^ sensing properties. 20 μL of a sonicated (10 min) 1 mg mL^−1^ solution of PBA in ethanol was then added onto the same grid. ODMR and MM measurements were then repeated in the same locations to register the paramagnetic response. Finally, TEM images were taken in the same locations to elucidate the locations and orientations of NPs of PBA and FND particles with respect to each other, and to correlate this information with NV^−^ sensing responses. Background signal from image stacks (areas where no FNDs or NPs of PBA were present – over vacuum) were subtracted from target signal to mitigate against the presence of background fluorescence.

## Result and discussion

### Bulk and surface magnetism

PBA materials have a wide range of magnetic properties^40^ including cryogenic photomagnetism for the cobalt iron (CoFe) PBA family^30,41^ and temperature dependant spin transitions.^42^ For example, the magnetic properties of the CoFePBAs can vary depending on Co and Fe ratios, as shown by Shimamoto *et al.* for XCoFePBA (X = Na, K, or Rb) where differing Co : Fe ratios were found to control the temperature and thermal hysteretic nature of a charge-transfer-induced spin transition (CTIST).^42^ In our magnetic studies, PBA showed no thermal hysteresis in the CTIST spin transition centred at 225 K (Fig. 2A), agreeing with observations made by Shimamoto *et al.*^42^ As we utilise NV^−^ sensing at room temperature (approximately 300 K), much higher than both CTIST and the magnetic ordering temperature, the PBA particles are in a paramagnetic, spin-disordered state with alternating paramagnetic Fe(iii)-LS and Co(ii)-HS centres.^43^

It is important to establish the surface composition of PBA NPs due to the high surface sensitivity of NV sensing, therefore X-ray Photoelectron Spectroscopy (XPS) was utilised. A survey scan confirms the presence of all expected elements (ESI File and Fig. S4†), and the high-resolution scan allows close examination of the Fe (Fig. 2B) and Co (Fig. 2C) 2p_3/2_ XPS regions. The Fe 2p_3/2_ region consists mainly of Fe(iii) signal (peak 2 at 710 eV) with a shoulder corresponding to an Fe(ii) (peak 3 at 709 eV), there is also a satellite, peak 1, at 712 eV.^44^ The Co peak consists of Co(ii) and Co(iii) signals present (peaks 5 at 786 eV, 6 at 783 eV respectively) and a satellite peak 4 at 790 eV.^45^ XPS analysis therefore is in agreement with the bulk SQUID measurements on the high temperature spin state of PBA showing presence of surface Fe(iii)-LS and Co(ii)-HS spin active centres. These paramagnetic metal centres should theoretically be the spin-active species which have dipole–dipole interactions with NV^−^ centres in the FND particles (statistically averaged fluctuating magnetic fields originate from paramagnetic spins of the transition metals). Surprisingly, EPR measurements at room temperature failed to detect any significant quantity of paramagnetic centres under these conditions (ESI File and Fig. S6†).^46^ The EPR silence of Co(ii) and Fe(iii) was observed for the related RbMn_*x*_Co_1−*x*_Fe PBA by Antal *et al.* who attributed absence of the EPR signal to charge-fluctuations between the high-temperature and low-temperature states causing rapid spin-lattice relaxation, broadening the EPR signal until it can no longer be observed.^47^

**Fig. 3:**
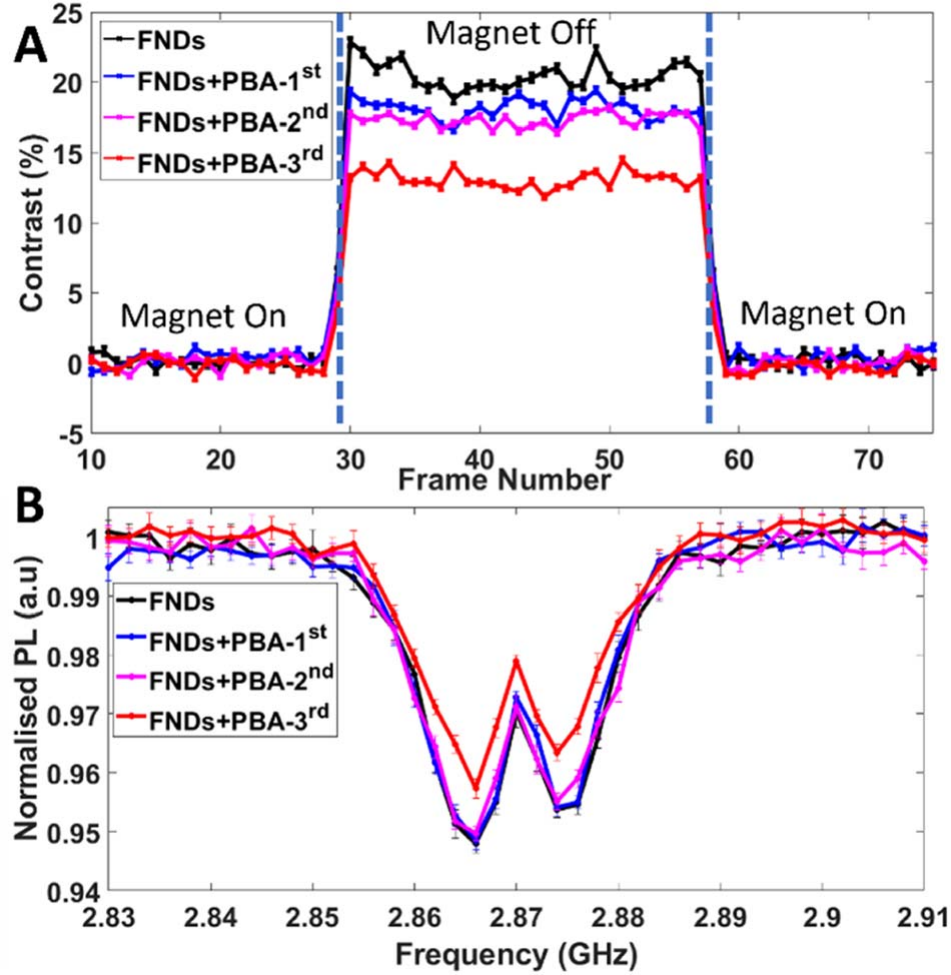
(A) MM and ODMR (B) traces for a series of PBA additions to a cluster of FNDs. The solid black line (FNDs) shows the optical response for the FND cluster before addition. The blue, magenta and red lines represent the 1st, 2nd and 3rd addition of the PBA material respectively. The 3rd addition of PBA shows a substantially larger PL contrast reduction compared to 1st and 2nd additions; this is due to more PBA material landing in the field of view in close proximity to FND cluster 2 upon addition (ESI File and Fig. S7† for optical images). Frame number here and throughout indicates an arbitrary time axis, this can be related to real time by multiplying by exposure times used (see Experimental section).

To demonstrate the feasibility of solid-state NV^−^ paramagnetic sensing with PBA NPs initial experiments were focused on clusters of PBA NPs deposited onto GCS covered with FNDs. The resulting PL contrast reduction was recorded as a function of accumulative deposition of PBA (1–3) (Fig. 3; optical images – bright field, fluorescence and an overlay, at each addition stage are shown in ESI File and Fig. S7†). A reduction in PL contrast from FNDs for both MM and ODMR measurements was observed, the extent of which is associated with the amount of PBA material deposited (Fig. 3A and B respectively). No evidence of residual magnetism is observed, in the absence of an applied field, when PBA particles are deposited, as indicated by an absence of Zeeman splitting of the ODMR double resonance. Co and Fe concentration (∼90 000 ppm) within PBA NPs give rise to similar NV contrast reduction observed in previous reports.^14,48^ A primary cause of this contrast change is considered to be the spin noise/fluctuations produced by the paramagnetic Fe(iii) and Co(ii) centres in PBA particles.^49^ Previous studies of paramagnetic samples using NV^−^ sensing schemes report a reduction in NV longitudinal spin relaxation time, *T*_1_, (the decay lifetime for NV initialised to a ground-state magnetic sublevel *e.g. m*_s_ = 0, to a thermal equilibrium mixture of states), owing to the presence of magnetic spin noise with a characteristic broadband spectral density, that can extend to the GHz range.^50–52^ Moreover, as the amount of paramagnetic material increases, so too does the NV relaxation rate, which is in part due to the concentration-dependent dipole–dipole interaction between the spin-active metal centres and NVs. In the context of the ODMR protocol used herein, reduction in *T*_1_ will lower the time-dependent probability of finding the NV in the *m*_s_ = 0 ground state and correspondingly a reduction in spin polarisation and ODMR contrast.^53,54^

The MM methodology employed here uses a large off-axis magnetic field to set the NV sensing quantization axis. Under these conditions *m*_s_ is no longer a good quantum number and the eigenstates of the spin Hamiltonian are described by superpositions of the *m*_s_ = 0 and ±1 spin sublevels leading to spin state mixing in both the ground and excited states (Fig. 1C).^55^ Experimentally this shortens the transverse relaxation time *T*_2_ and increases the mean probability for non-radiative intersystem crossing (ISC) transitions for all spin states from the excited state to the metastable level, leading to a reduction in NV PL. The presence of proximal paramagnetic spin centres further reduces the efficiency of spin polarization and the excited level lifetime, as observed as reduced MM contrast.^9,56,57^

The above reported measurements on interactions between FNDs and clusters of PBA NPs yields averaged information which lacks precise details of the exact relationship between the interacting species, such as interparticle separation or amount (local concentration) of interacting PBA NPs. Spatial resolution beyond that of light microscopy is required to visualise interaction of FNDs and PBA NPs. Here, TEM imaging was employed to provide a path towards quantitative understanding of these interactions, down to the nanoscale level.

### Prussian blue analogue and fluorescent nanodiamond transmission electron microscopy

Drop casting of freshly prepared suspensions of PBA and FND particles onto a TEM grid allows formation of dispersions where both particles are mixed on the surface of amorphous carbon film (Fig. S9A†). Despite having a similar contrast in bright-field TEM, FNDs and PBA NPs can be clearly distinguished by their shapes. The size and morphology of PBA NPs depend on precursor addition rates,^58^ surfactant modification^58^ and surface functional groups.^59^ In our materials they appear as pseudo spherical/cuboctahedral in shape due to isotropic propagation of the face centred cubic (fcc) lattice during particle growth. FND particles have flat edges with acute angles, characteristic to the diamond lattice. The average size of PBA and FNDs were determined as 120 ± 7 nm and 110 ± 5 nm from TEM image analysis, and hydrodynamic diameters 170 ± 60 nm and 160 ± 60 nm from dynamic light scattering (DLS), respectively (ESI File and Table S1†). TEM imaging allows determination of the interparticle separation on the grid in the specific locations (Fig. S9B and C†) that can be re-located in the light microscope due to the alphanumerical navigation grid (large field of view light and electron microscopy images ESI, Fig. S13†). Furthermore, HRTEM and selected area electron diffraction (SAED) determines the atomic lattice of PBA where the *d*-spacings are indexed and correlated with PXRD (ESI File and Fig. S1†) to confirm the crystal phase (Fig. S9D and E† respectively) for fcc lattice of PBA NPs.^60,61^ EDX spectroscopy confirms the presence of expected metals and ratios in PBA particles Co, Fe and Rb (ESI File and Fig. S14†). Without care, the e-beam is known to be an invasive probe that can change both structure and composition of nanomaterials during imaging through a number of different mechanisms (*e.g.* direct knock-on damage, radiolysis and heating).^62^ Control measurements were carried out to evaluate the impact of the 200 kV e-beam irradiation on FND and PBA particles (ESI File and Fig. S10† for full e^−^ beam irradiation discussion).

### Non-integrated correlative light-electron microscopy nitrogen vacancy sensing on TEM finder grids

**Fig. 4:**
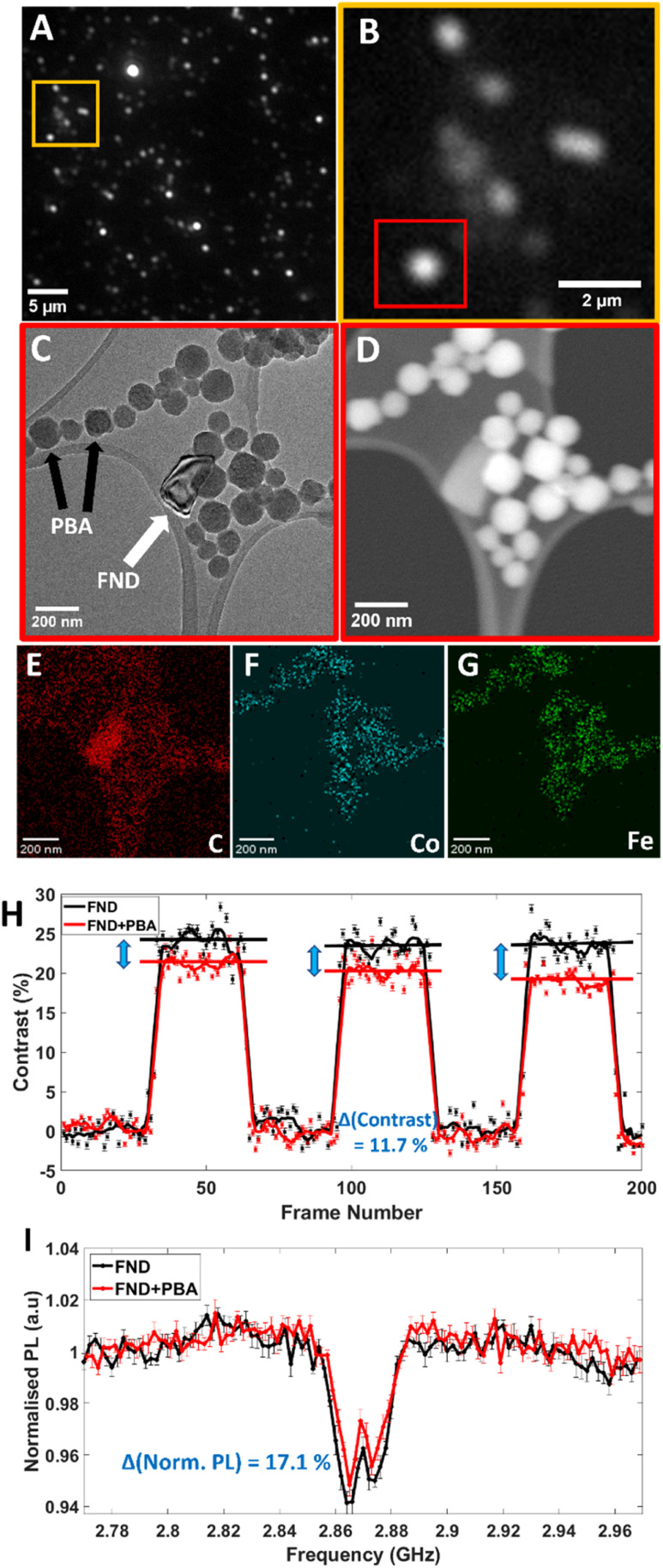
IL-CLEM NV^−^ sensing of PBA NPs. (A) PL image of drop cast FNDs on a TEM grid. (B) Zoomed-in area of (A) showing the FND of interest. (C) and (D) TEM and STEM images respectively indicating the same area of interest highlighted in (B). Here, we see the exact FND-PBA interacting particles which give the target localised paramagnetic PL response. (C) indicates the FND of interest by a white arrow. (E–G) STEM-EDX mapping of carbon, cobalt and iron respectively. (H) Background subtracted MM trace and (I) ODMR spectrum of the interacting FND-PBA particles. The black trace shows the contrast from the isolated FND particle before PBA addition and the red trace shows the contrast after PBA NPs are added. For MM, raw data is shown alongside averaged data for each ‘on’ and ‘off’ application of the external magnetic field. Error bars for the averaged data are shown and were calculated using simple sum standard deviation analysis. For both MM and ODMR, the quantitative difference between the FND and FND + PBA signal is shown in blue (%) – the method for calculating this difference is in Fig. S21 of the ESI.†

The workflow of our CLEM NV sensing approach designed to sense paramagnetism of PBA NPs from single FNDs or small clusters, is indicated in the following steps. First, background ODMR and MM PL spectra were recorded, and positions of small clusters or single FND particles were identified *via* visual inspection of PL image stacks. Clusters of FNDs can be imaged with light microscopy, but individual FND particles cannot be resolved due to the diffraction limit (Fig. 4A and B). Due to their lower emission, single particles appear as spots of lower brightness alongside bright, large clusters of FNDs. Next, the same area was imaged by TEM, confirming the presence of single FND particles and their sizes and shapes. Then, PBA NPs were drop cast on the same TEM grid which allowed the formation of FND-PBA pairs separated by different distances. At this stage a second round of PL spectra were recorded to ascertain the paramagnetic response. Finally, TEM imaged the interacting FNDs and PBA NPs (Fig. 4C and D). There are different methods one can use to ensure the exact same area is located when going to and from light and electron microscopy.^63^ Here, the pattern of the carbon film can be used as an identifying marker with features unique to only that area. Typical evidence for a PL spot seen in optical microscopy being a single FND is obtained from IL-TEM images (ESI File and Fig. S17†). The change in PL contrast observed for the FND in close contact with the PBA NPs is typical of the effect of unpaired electron spin noise on the emission of negatively charged NVs. Paramagnetic sensing is evidenced by PL contrast reduction in MM (Fig. 4H) and in ODMR (Fig. 4I). Scanning transmission electron microscopy (STEM)-EDX mapping was performed to confirm relative positions of interacting NPs (Fig. 4E–G, high carbon signal can be seen for the FND particle. PBA particles indicate spin active cobalt and iron). Furthermore, the STEM imaging contrast which is highly dependent on atomic number (*z*) and scales as approximately proportional to *z*^1.8^, as opposed to bright-field TEM which scales as approximately 
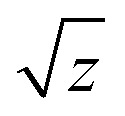
, can be used to better differentiate metal-containing PBA particles from metal-free FNDs allowing determination of their relative positions.^64^

For single FND particles or clusters not in proximity to any spin active material, before addition and after addition of PBA to the grid, NV^−^ sensing response remains nearly identical, in contrast to FNDs close to PBA particles which show a measurable change (Fig. S18B and A† respectively). This evidences the capability of our reported methodology to detect the presence or absence of a paramagnetic response.

**Fig. 5:**
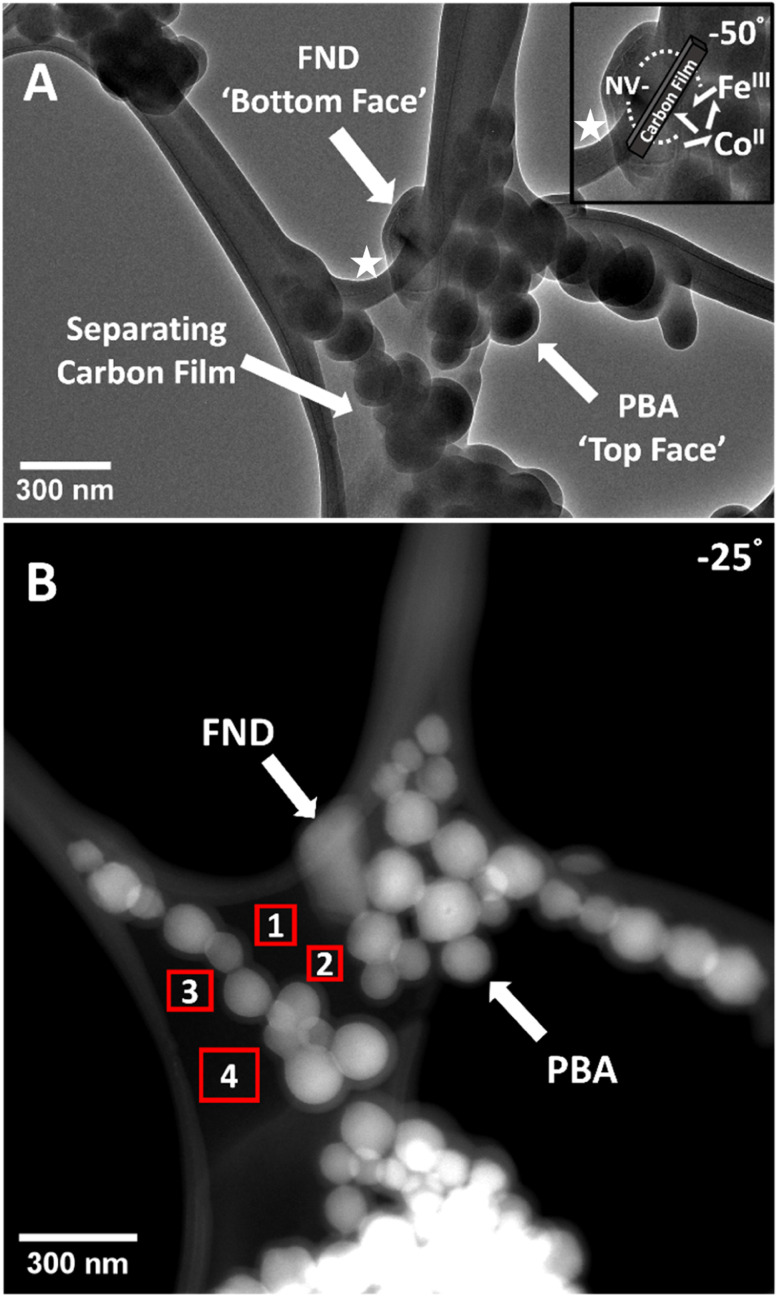
(A) BF-TEM tilt series image (−50°, top right, around the *x*-axis of the stage holder) of the interacting single FND and PBA NPs. The single plate-like FND particle is on the opposite side of the carbon film to the spherical PBA NPs. Videos of tilt series both in BF-TEM and DF-STEM are the ESI Section.† (note that surfaces of NPs became coated with an amorphous deposit after SEM measurements, taken after all NV sensing measurements, details are in ESI File and Fig. S17†). Inset in A, a zoomed-in image of the spin-active interacting particles (area shown using a white star) highlighting the theoretical dipole–dipole interactions between randomly oriented paramagnetic Fe^III^ and Co^II^ unpaired electrons (white arrows) and NV^−^ centres through the carbon film. (B) DF-STEM image (−25°, top right, around the *x*-axis of the stage holder) showing selected areas of EELS thickness analysis. Red boxes indicate the area in which STEM-EELS analysis was taken. EELS measurements were taken at 0° tilt but here a −25° image is used to better illustrate areas of analysis. Carbon film thickness measured in area 1 11 nm, area 2 12 nm, area 3 13 nm and area 4 13 nm. Averaging over these areas (and the other areas away from target as shown in ESI File and Fig. S20† – 13 nm and 18 nm) gives 13 ± 5 nm (*x̄* ± 2*σ*).

Standard TEM images are 2D projections of a 3D system. To understand the nature of the single FND through space interaction with PBA NPs (Fig. 4C and D) in detail as well as the extent of the ODMR and MM response, TEM and STEM tilt series were performed around the *x*-axis −50 to 50° (Fig. 5 and Video files† – see additional files). This revealed the single FND particle was on the opposite side of the carbon film to the PBA NPs, hence separated by a longer distance than it may appear from standard 2D TEM micrographs. This highlights the importance of sample tilt in CLEM measurements, as it provides more accurate information on FND-PBA separation in 3D space. We apply this methodology to two specific cases where FND-PBA particles seem to form direct physical contact based on 2D projections in TEM, but they had a detectable difference in NV^−^ PL response. It was not until studying 3D TEM tomography, that we observed they had very different interactions, with FND and PBA being on the opposite sides of the film in one case, and in direct physical contact in the other case (ESI File and Fig. S22† and Video files). TEM videos of the sample tilt demonstrate clearly how the particles move relative to one another: if a pair of FND-PBA particles move together through rotation, they are likely to be on the same plane (*i.e.* same side of the carbon film), if the FND-PBA particles move away from one another through rotation, they are likely to be on different sides of the carbon film. Next is the question of thickness of the amorphous carbon support film. It is expected to be in the range of 10–20 nm. To measure this more accurately and locally, STEM-EELS was applied for several locations around the area of interest, as illustrated in Fig. 5B. Thickness was also measured in areas away from the area of interest to act as a control in case several iterations of different imaging conditions affect the result (Fig. S20†). There were no considerable differences in film thickness around the target area compared to locations away from target in different grid positions with an average film thickness of 13 ± 5 nm (*x̄* ± 2*σ*). The precise knowledge of the thickness of the supporting film improves the accuracy of FND-PBA separation measurements and demonstrates that NV^−^ sensing of paramagnetic PBA particles can be achieved through an amorphous carbon film barrier between a single FND and PBA NPs. Significantly, the ability to sense through a thin film opens new applications for this methodology, including through-space quantum technologies, such as local probing arrays of encapsulated magnetic units optically through nanotube walls.^65,66^

**Fig. 6:**
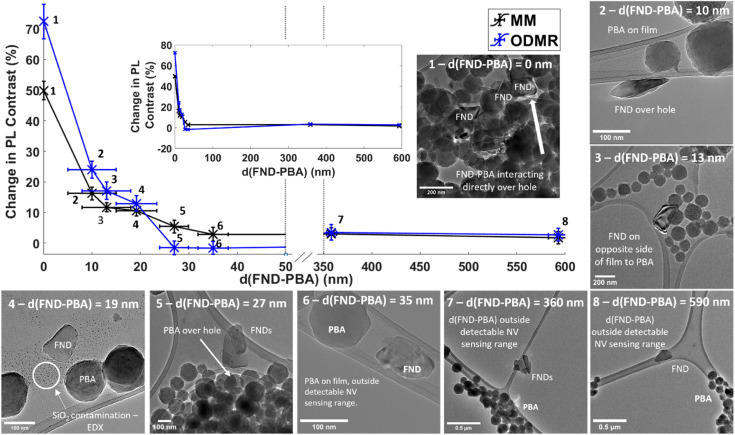
Change in FND PL contrast (%) as a function of shortest FND-PBA interparticle edge-to-edge separation, d(FND-PBA). Inset shows a plot of continuous *x*-axis. As NV sensing protocol used herein is a surface sensitive technique, distance is measured in 2D from TEM images between shortest edges of the FND and PBA (except for data point 3 which is measured from EELS), PL is measured from the whole FND(s). A table of extracted numerical values of contrast change for both NV sensing schemes, MM and ODMR, can be found in ESI, Table S3.† Individual ODMR spectra and MM traces can also be found in the ESI, Fig. S21.† TEM micrographs of each area, 1–8, show positions and orientations of paramagnetically interacting or non-interacting FND-PBA particles. Location 4 had traces of silica contamination on the carbon support as evidenced by EDX spectroscopy (ESI File and Fig. S23† for more information). For justification of distance measurements and error see ESI, Fig. S24.†

To study the FND and PBA particle separation dependent NV^−^ based sensing scheme used herein, a number of individual FNDs and small clusters were identified with TEM and their NV response extracted (Fig. 6). Individual FNDs/small clusters were chosen as a control (FND particle packing in a large cluster might affect available surface area for interaction), and separation was defined as the shortest edge-to-edge distance between FND and PBA particles (majority of modulated NV^−^ signal will be measured from surface or near surface NVs in FNDs due to presence of paramagnetic transition metal centres on the surface of PBA particles respectively, following that the dipole–dipole interaction strength varies as an inverse cube of distance). Tilt series analysis was conducted for locations where both FND and PBA particles, from 2D inspection, were overlapping/in direct contact on the carbon film (or in very close proximity), hence the value of vertical separation can be significant for the interparticle separation in 3D. Only location 3 in Fig. 6 has FND and PBA particles both on the carbon film but on opposite sides. Location 1 shows a large cluster of PBA NPs positioned on a small cluster of FNDs. A large proportion of the FND surface is covered in PBA (direct contact is expected as some FND and PBA particles are positioned over holes/vacuum of the TEM grid, therefore distance ∼0 nm away from the FND surface is assigned) which gives a large PL contrast reduction in both MM and ODMR. Values of contrast reduction (%) for both NV sensing schemes in all locations in Fig. 6 can be found in the ESI, Table S3.† Location 2 shows a FND particle hanging over the edge of film which is approximately 10 nm away from the closest PBA NP edge. Location 3, previously described, has FND PBA particles that are separated by amorphous carbon film with a thickness of 13 nm measured by EELS. Location 4 shows a single FND particle in close proximity to a PBA particle with a separation of 19 nm, giving an NV sensing response similar to location 2 and 3, relatively small but significant. Location 5 shows two FND particles next to a cluster of PBA particles that are hanging over the edge of a film hole, separated by approximately 27 nm. At this distance, no significant contrast reduction was observed. Locations 6, 7 and 8 show FND-PBA separations of 35, 360 and 560 nm respectively, all showing negligible paramagnetic sensing response. Fig. 6 depicts the rapid initial change of NV contrast with FND-PBA separation followed by a plateau. With the signal to noise ratios achieved using our experimental system, the maximum useable sensing range of the PBA particles lies between locations 4 (19 nm) and 5 (27 nm), closely correlated with previously reported NV proximity sensing.^67–69^ (ODMR spectra and MM traces of all areas in Fig. 6 can be found in ESI File and Fig. S21†).

**Fig. 7:**
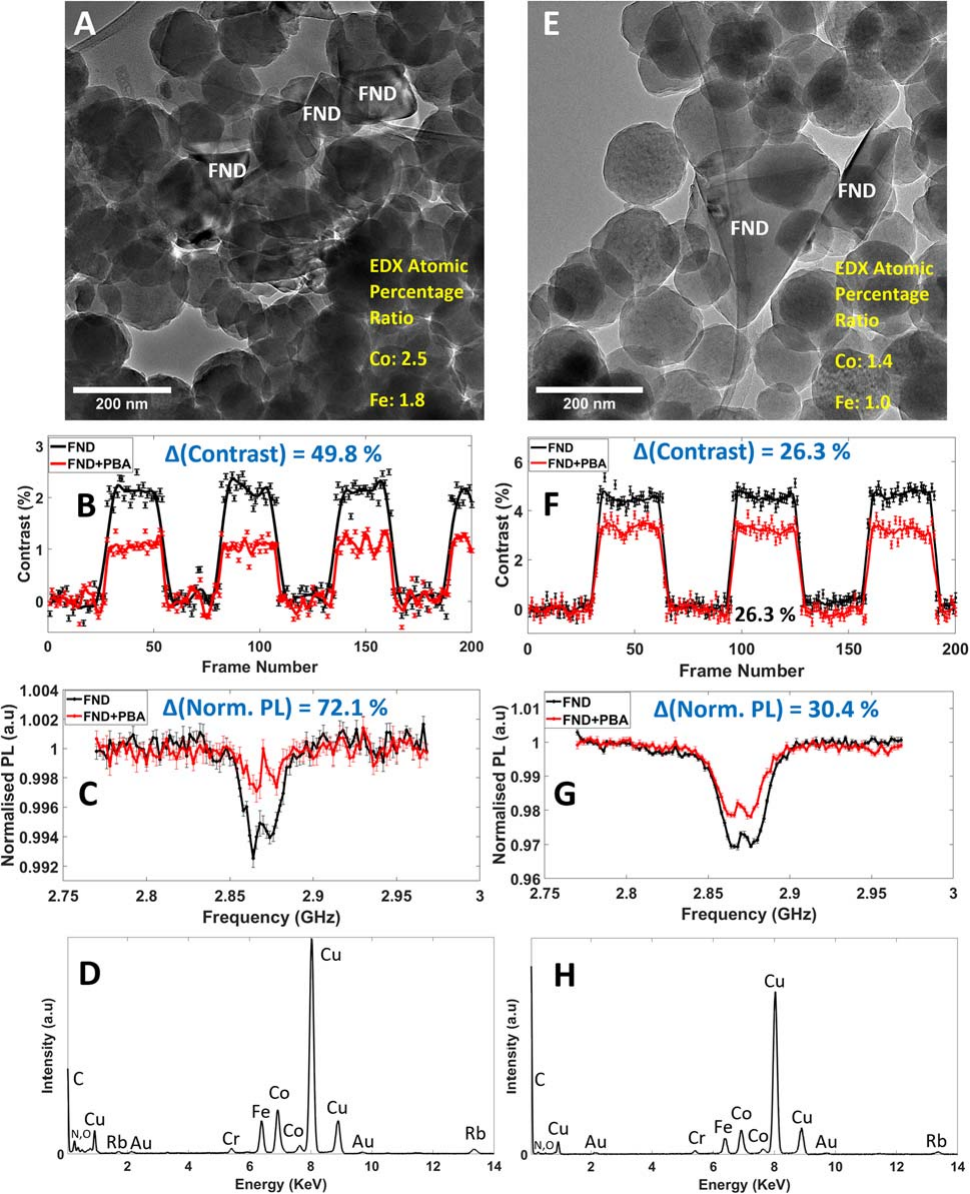
Local EDX analysis of two locations TEM micrographs (A) and (E) with FND-PBA particles in direct contact (distance between FND and PBA is assumed to be 0 nm). NV sensing response for both MM and ODMR, (B) and (C) for location (A), (F) and (G) for location (E) respectively is shown. (D) and (H), local EDX spectra of locations (A) and (E) respectively. EDX atomic percentage ratios for spin active Co and Fe is shown in inset of (A) and (E), ratio normalised in both from Fe (1.0) in spectra (H). EDX spectra (spot size of electron beam during measurement) were obtained roughly over the entire areas shown in (A and E).

Another factor to consider, responsible for FND PL contrast reduction, is local concentration, *i.e.* the number density of particles with spin-active centres in close proximity to NV centres within FNDs which needs to be taken into consideration as well as FND-PBA interparticle distance (Fig. 7). Here, two locations are chosen (primarily over holes of the film to mitigate separating film effects) where FND and PBA particles overlap (interparticle FND-PBA distance is assumed 0 nm in both cases). The two locations show significantly different NV sensing responses although FND-PBA interparticle distance is assumed identical. The justification for location Fig. 7A having larger PL contrast reduction (Fig. 7B and C) compared to location Fig. 7E (Fig. 7F and G) is down to the surface coverage of PBA spin-active particles on or close to the FND surface. Fig. 7A has FNDs that are in contact with a very large cluster of PBA NPs. Fig. 7E visually has fewer interacting particles (a large proportion of one of the FND faces is not in contact with surrounding PBA) and therefore has a smaller NV response. Local EDX measurements of atomic percentage ratios of spin active Co and Fe, reflecting the number density of particles, affects the extent of the NV response (Fig. 7A and E inset, D and H for EDX spectra). Greater Co and Fe atomic percent is calculated for location A (Co: 2.5, Fe: 1.8) compared to location E (Co: 1.4, Fe: 1.0).

It is therefore important when attempting to quantify NV sensing responses, within a certain material, to combine both distance between interacting NPs and amount of proximal spin active particles close to FNDs. We demonstrate both these effects and advance a methodology towards quantitative solid-state NV sensing, down to the nanometre length scales.

## Conclusions

We demonstrated that paramagnetic spin fluctuations of transition metals within a NP can be effectively detected directly by NV^−^ defects in single FND particles. PL contrast reduction of FNDs, evidenced by sequential decreases in ODMR and MM contrast, correlates with the amount of paramagnetic PBA (a cobalt iron Prussian blue analogue) NPs in close proximity to FNDs. We harnessed this effect down to the single-particle level using non-integrated correlative light-electron microscopy methodology to monitor changes in ODMR and MM signals as a function of edge-to-edge FND-PBA interparticle separations and local concentration (number density of close proximity interacting FND-PBA particles), using TEM finder grids as an experimental platform. Detailed analysis of spin active NPs of PBA by SQUID magnetometry, EPR, XPS and EDX spectroscopies prior to coupling to FNDs on the grid, and careful control of e-beam fluence to avoid damage, allowed us to conclude that paramagnetic dipole–dipole interactions between NV^−^ centres in FNDs and spin fluctuations from transitional metals in PBA NPs can be detected at interparticle separations on the order of 10 nm (the maximum detectable interparticle separation, with current signal to noise ratios, was found to lie between 19 nm and 27 nm). Tilting the sample grid in TEM allowed us to enhance the accuracy of inter-particle separation determination and elevated CLEM at nanoscale into 3D space. Significantly, paramagnetic dipole–dipole interaction was shown to take place through a 13 nm film of amorphous carbon separating FND and PBA on the grid, evidenced by TEM tilt series and EELS measurements. Ongoing studies will aim to build on single-particle, identical-location sensing methodology by extending this approach to switchable magnetic systems, such as spin crossover (SCO) and photomagnetic materials. Additionally, the ability to sense through thin films will be exploited to study encapsulated arrays of magnetic species inside nanotubes.

Overall, the advancement in current methodologies and workflow reported herein that combine the power of two local probes, NVs and electron beam, provide a pathway for quantitative nanoscale sensing of functional and structural information with tremendous potential to accelerate the development and implementation of quantum technologies, such a memory storage, computing or molecular switching nanodevices.

## Data availability

Data is available on request to corresponding authors.

## Conflicts of interest

There are no conflicts to declare.

## Supplementary Material

NA-005-D3NA00155E-s001

NA-005-D3NA00155E-s002

NA-005-D3NA00155E-s003

NA-005-D3NA00155E-s004

NA-005-D3NA00155E-s005
