# Supplementary material for: Nitrogen vacancy defects in single-particle nanodiamonds sense paramagnetic transition metal spin noise from nanoparticles on a transmission electron microscopy grid
Source: Nanoscale Adv. 2023 Aug 18;5(23):6423–34. doi: 10.1039/d3na00155e (PMC10662216; doi:10.1039/d3na00155e)
Supplement: NA-005-D3NA00155E-s001 [file NA-005-D3NA00155E-s001.pdf]

Electronic Supplementary Material (ESI) for Nanoscale Advances.
This journal is © The Royal Society of Chemistry 2023

Nitrogen Vacancy Defects in Singe-Particle Nanodiamonds Sense Paramagnetic Transition Metal Spins in Nanoparticles on a Transmission Electron Microscopy Grid

Instructions for EELS_sample_thickness.m

This script is a modified version of EELS_fit_analysis.m.[1] For more detailed instructions consult the original publication. This script uses the trapezoid method to calculate the integral of the zero-loss peak and plasmon of an EELS spectrum, then returns the thickness of the area using the log ratio method.[2] The inelastic mean free path is estimated using the free electron formula.[2] It is worth noting here that this code does not account for plural electron scattering and therefore is only valid for thin samples.

Importing data is the same as for EELS_fit_analysis.m. Briefly, the EELS spectrum should be saved as an .msa file, which should be placed in the same folder as EELS_sample_thickness.m, or the path defined to the .msa file. The user defined variables should then be set:

```
beamEnergy =200e3;
collectionAngle = 100e-3;
plasmonEdge = 24;
PlasmonEnd =50;
ZLPstart=0;
ZLPend=18;
shift=9;
startData = 0;
endData = 300;
```

Where beamEnergy is the energy of the electron beam in eV, collection angle is the semi-angle of collection in rad, plasmonEdge is the maximum energy of the plasmon, plasmonEnd is the end of the plasmon, ZLPstart is the start of the zero-loss peak, ZLPend is the end of the zero-loss peak, shift is an integer that allows MATLAB to integrate over positive numbers only, startData is the start of the EELS data, and endData is the end of the EELS data. For example, if the zero-loss peak starts at -6 and ends at 10: ZLPstart = -6, ZLPend = 10, shift = 6.

The spectrum is then fitted using a Gaussian function, which will allow for the zero-loss peak and plasmon to be integrated. The number of terms in the fit can be variable from 1 to 8 using ‘gauss1’ to ‘gauss8’. To fit using other functions, see Fung et al..[1]

```
[f,gof,output] = fit(xdata2,ydata2,'gauss8');
residuals = ydata2 - f(xdata2);
fitoptions = fitoptions(f);
```

The zero-loss peak and plasmon are then integrated using the user-defined limits and the above fit. These are returned as ZLPint and PlasmonInt, and the ratio of the two is returned as IntRatio.

The free electron formula is then used to estimate the inelastic mean free path of electrons in the sample, returned as MFP. This can be replaced with an exact mean free path if it is known for your material.

The ratio of the integrals and the mean free path is then used to calculate the thickness of the sample in nm, returned as thicknessnm.

References

1) Fung et al., *Ultramicroscopy*, 2020, **217**, 113052
2) Brydson, Rik. *Electron Energy-Loss Spectroscopy*. Oxford: Taylor and Francis, 2001.
