# Supplementary material for: Nitrogen vacancy defects in single-particle nanodiamonds sense paramagnetic transition metal spin noise from nanoparticles on a transmission electron microscopy grid
Source: Nanoscale Adv. 2023 Aug 18;5(23):6423–34. doi: 10.1039/d3na00155e (PMC10662216; doi:10.1039/d3na00155e)
Supplement: NA-005-D3NA00155E-s005 [file NA-005-D3NA00155E-s005.pdf]

Electronic Supplementary Material (ESI) for Nanoscale Advances.
This journal is © The Royal Society of Chemistry 2023

Supplementary Information

Nitrogen Vacancy Defects in Singe-Particle Nanodiamonds Sense Paramagnetic Transition Metal Spins in Nanoparticles on a Transmission Electron Microscopy Grid

Bradley T. Flinn, [a] Valentin Radu, [b] Michael W. Fay, [c] Ashley J. Tyler, [b] Jem Pitcairn, [a] Matthew J. Cliffe, [a] Benjamin L. Weare, [a] Craig T. Stoppiello, [d] Melissa L. Mather*, [b] Andrei N. Khlobystov*[a,c].

Contents

List of Figures 1

List of Tables 2

List of Figures

S1. Powder X-ray diffractogram for $Rb_{0.5}Co_{1.3}[Fe(CN)_6]\cdot 3.7H_2O$ = PBA 3

S2. Size distributions of fluorescent nanodiamonds (FNDs) and PBA from transmission electron microscopy (TEM) analysis 3

S3. Isothermal magnetisation M(H) measurements at 2 K for PBA 5

S4. Survey scan X-ray photoelectron spectrum (XPS) for PBA 6

S5. Attenuated total reflectance infrared (IR) spectrum for PBA 6

S6. Electron paramagnetic resonance (EPR) spectrum of PBA 7

S7. Fluorescence, brightfield and overlay images of drop cast FNDs and PBA 8

S8. Optically detected magnetic resonance (ODMR) spectra and magnetic modulation (MM) traces for ND cluster 1 and 2 as shown in S7 9

S9. TEM imaging of PBA NPs and FNDs dispersed onto a Copper lacey carbon finder grid with d-spacing and selected area electron diffraction (SAED) analysis 10

S10. Effects of the transmissions electron microscopy (TEM) beam on nitrogen vacancy (NV) sensing MM traces 11

S11. Effects of TEM beam on fluorescence of FNDs. Fluorescence images and TEM micrographs before and after high electron flux are shown 13

[a]School of Chemistry, University of Nottingham, University Park, Nottingham, NG7 2RD, UK.
[b]Optics and Photonics Group, Faculty of Engineering, University of Nottingham, University Park, Nottingham, NG7 2RD, UK.
[c]Nanoscale and Microscale Research Centre, University of Nottingham, Nottingham, NG7 1QL, UK.
[d]Centre for Microscopy and Microanalysis, University of Queensland. St Lucia, 4072, Australia.

*[*melissa.mather@nottingham.ac.uk](mailto:melissa.mather@nottingham.ac.uk); andrei.khlobystov@nottingham.ac.uk*

Electronic Supplementary Information (ESI) available: [Supplementary information can be found at xx]. See DOI: 10.1039/x0xx00000x

S12. Low electron flux and high electron flux TEM micrographs of PBA nanoparticles, including beam damage product analysis 15

S13. Low magnification TEM and optical images of a TEM finder grid 16

S14. Energy dispersive X-ray (EDX) spectrum of PBA 17

S15. ODMR spectrum for the non-magnetically interacting FND particles shown in Figure S18 17

S16. Description of the background subtraction technique used for NV sensing analysis 18

S17. Non-integrated correlative light-electron microscopy imaging for FND relocation 19

S18. MM traces for single FND particles that gives a paramagnetic NV sensing response and no response, with corresponding TEM images confirming the presence or absence of spin active PBA NPs 20

S19. BF-TEM tilt series imaging of the interacting FND and PBA NPs described in Figure 4 of the main text 21

S20. Scanning TEM electron energy-loss spectroscopy (STEM-EELS). An example of EELS carbon film thickness measurements 22

S21. ODMR spectra and MM traces for all FND areas shown in Figure 6 of the main text 23

S22. TEM tilt series and NV sensing analysis explaining the importance of 3D visualisation with respect to NV sensing analysis 25

S23. EDX spectrum of common Silica contamination on the TEM grid amorphous support film 26

S24. Schematic explanation of the two different distance measurement protocols, with errors, used in Figure 6 of the main text 27

List of Tables

S1. Comparison of numerical TEM and dynamic light scattering (DLS) particle size data for FNDs and PBA, zeta potential measurements are also provided 4

S2. EDX atomic percentage ratios of all non-carbon elements in PBA at different electron flux to monitor electron beam sensitivity 14

S3. A table of extracted numerical values of contrast for both NV sensing schemes, MM and ODMR, as a function of PBA separation for 7 locations imaged in the main text 26

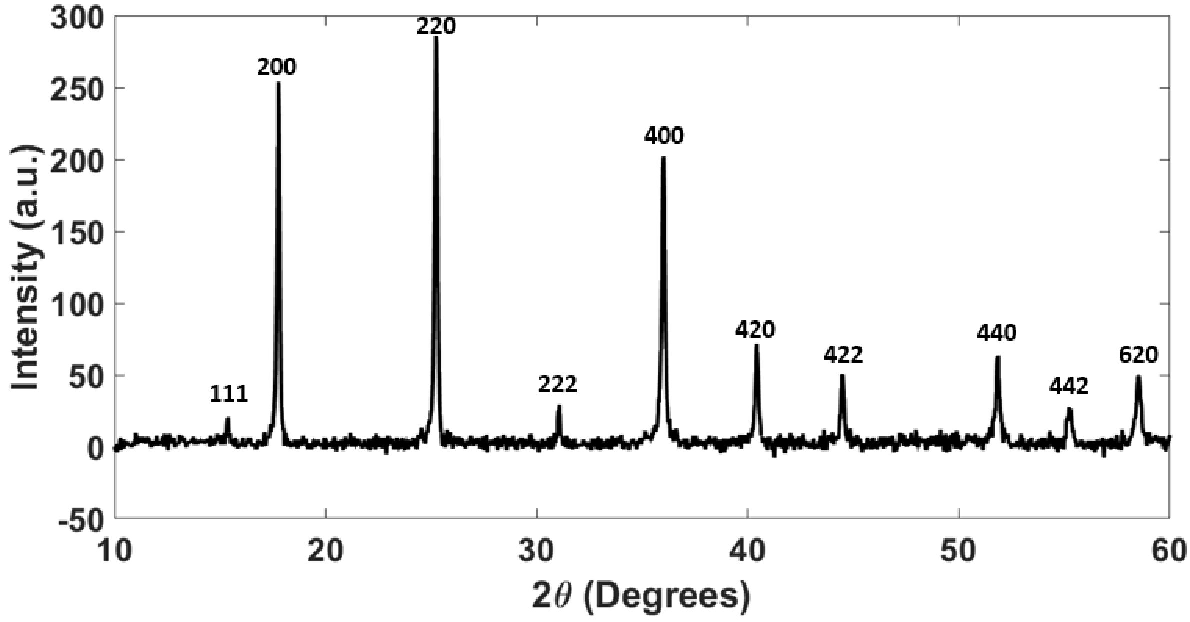

Figure S1. Powder X-ray diffractogram for $Rb_{0.5}Co_{1.3}[Fe(CN)_6]\cdot 3.7H_2O$ = PBA. Peak values (°) and corresponding index planes: 15.92 (111), 17.78 (200), 25.21 (220), 30.02 (222), 36.01 (400), 40.43 (420), 44.46 (422), 51.82 (440), 55.20 (442), 58.50 (620), in line with previous reports.[1]

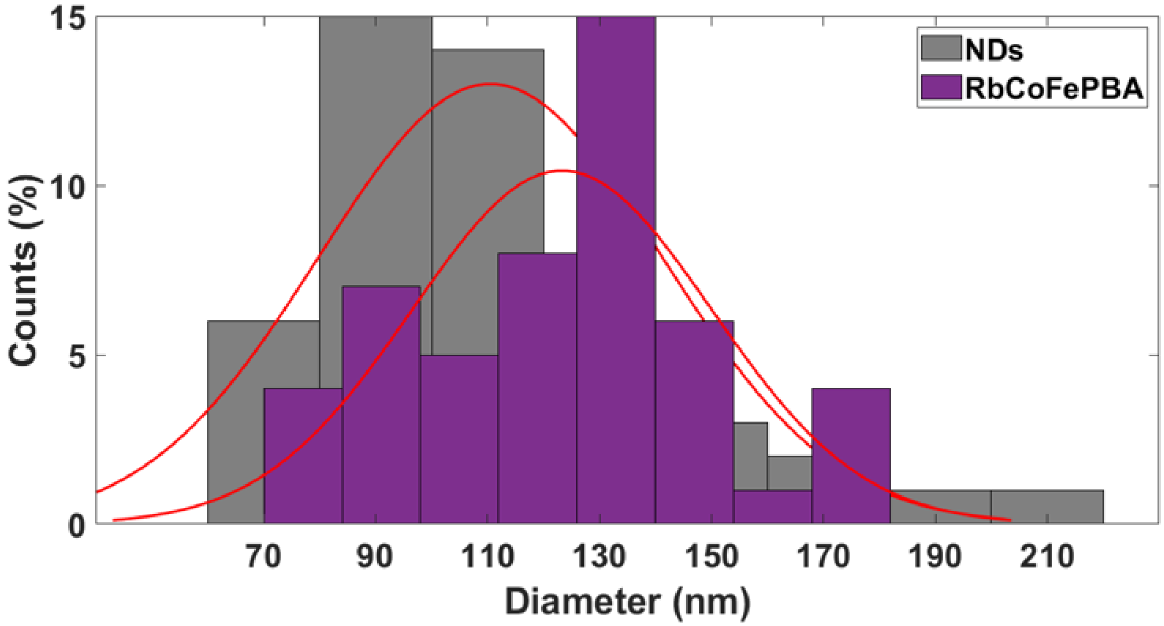

Figure S2. Size distributions of FNDs (grey) and PBA from TEM analysis. Sizes were manually measured using the ImageJ software for 50 of each particle. For PBA particles 3 random diameters were chosen for each particle and averaged. For FNDs, a straight line from 3 points of sharp faceted triangular particles to the opposite side, was chosen and averaged. The centre of the distribution for FNDs was 110 $\pm$ 5 nm. PBA was 120 $\pm$ 7 nm.

Table S1. Hydrodynamic diameter and zeta potential data for the FND and PBA nanoparticles studied in this work. DLS and zeta potential errors automatically calculated as a standard deviation and errors from TEM images manually measured from ImageJ software is assumed to be the resolution limit of the microscope (human error is smaller than resolution limit). Hydrodynamic size from DLS is larger than TEM sizes due to the over estimation of DLS measurements (In a dispersion where there is a distribution of particle size, larger size particles scatter light more greatly and are given more weight in size binning, leading to an overall larger size given).[2]

Material	Dynamic Light Scattering hydrodynamic diameter of particles (nm)	TEM Particle Size (nm)	Zeta Potential (mV)
FND	160 ± 60	110 ± 78	-37 ± 4.3
PBA	170 ± 70	120 ± 70	-34 ± 3.8

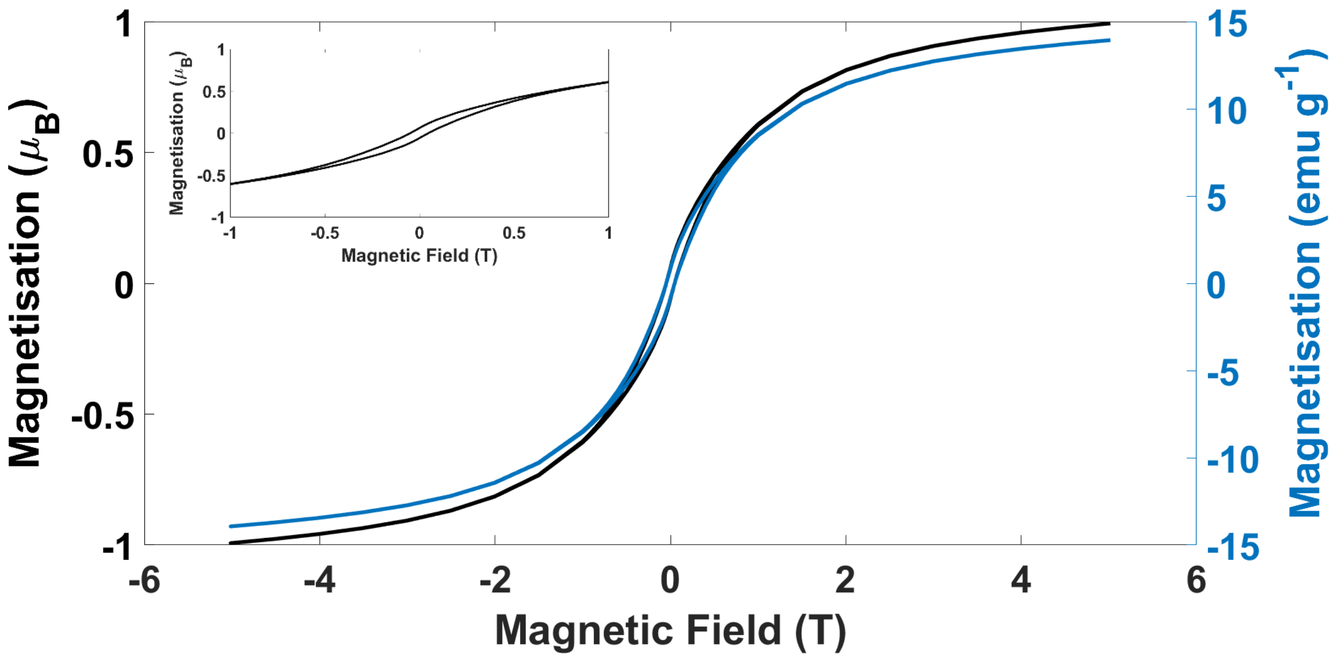

Figure S3. Magnetisation vs field, M(H), of PBA at 2 K. An inset shows the low field behaviour (−1 to 1 T) which makes clear the soft ferrimagnetic behaviour below the ordering temperature (14.5 K), consistent with a negative Weiss constant (main text, **Figure 2**). At saturation magnetisation, $M_{sat} = 1\ \mu_B$. Assuming g =2, <S> = 0.5 per formula unit.[3] This gives a spin concentration, <S>/V = 2 nm^{-3}. At room temperature, metal centres will consist of paramagnetic S=3/2 Co(II)-HS and S=1/2 Fe(III)-LS states (**Figure 2** in the main text). [3-6]

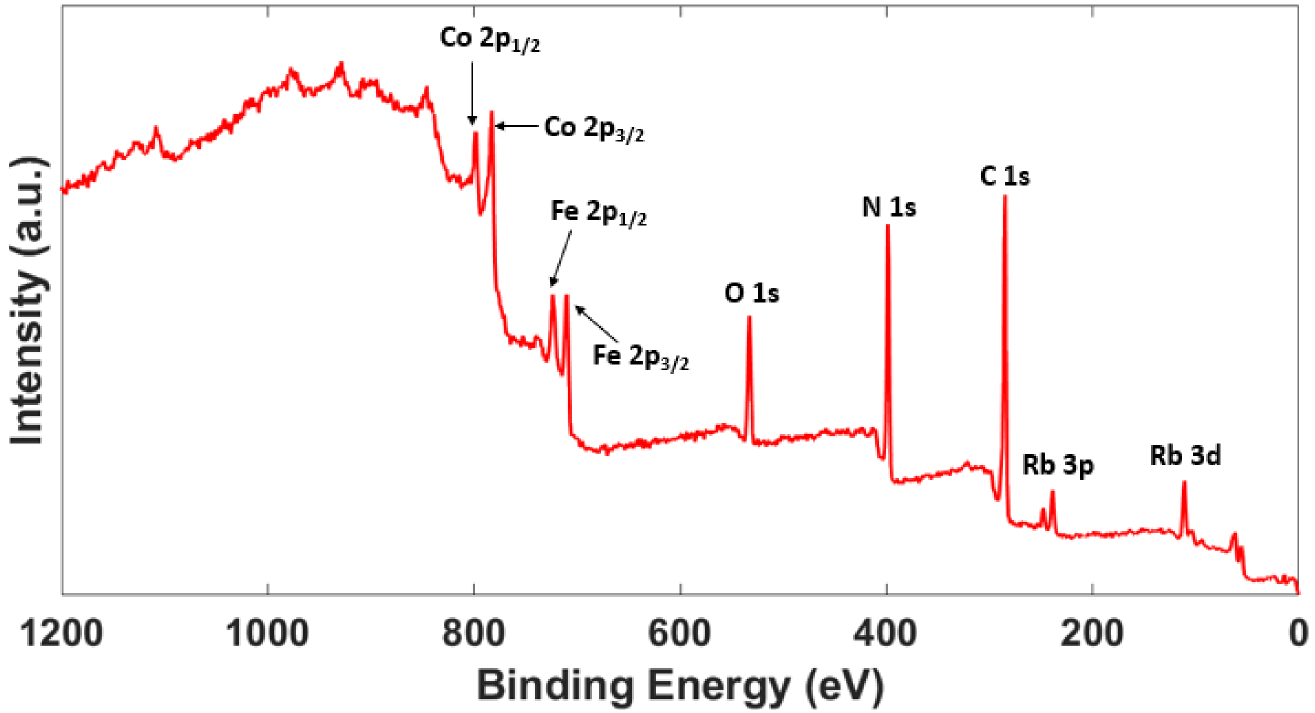

Figure S4. Survey-scan X-ray photoelectron spectrum of PBA.

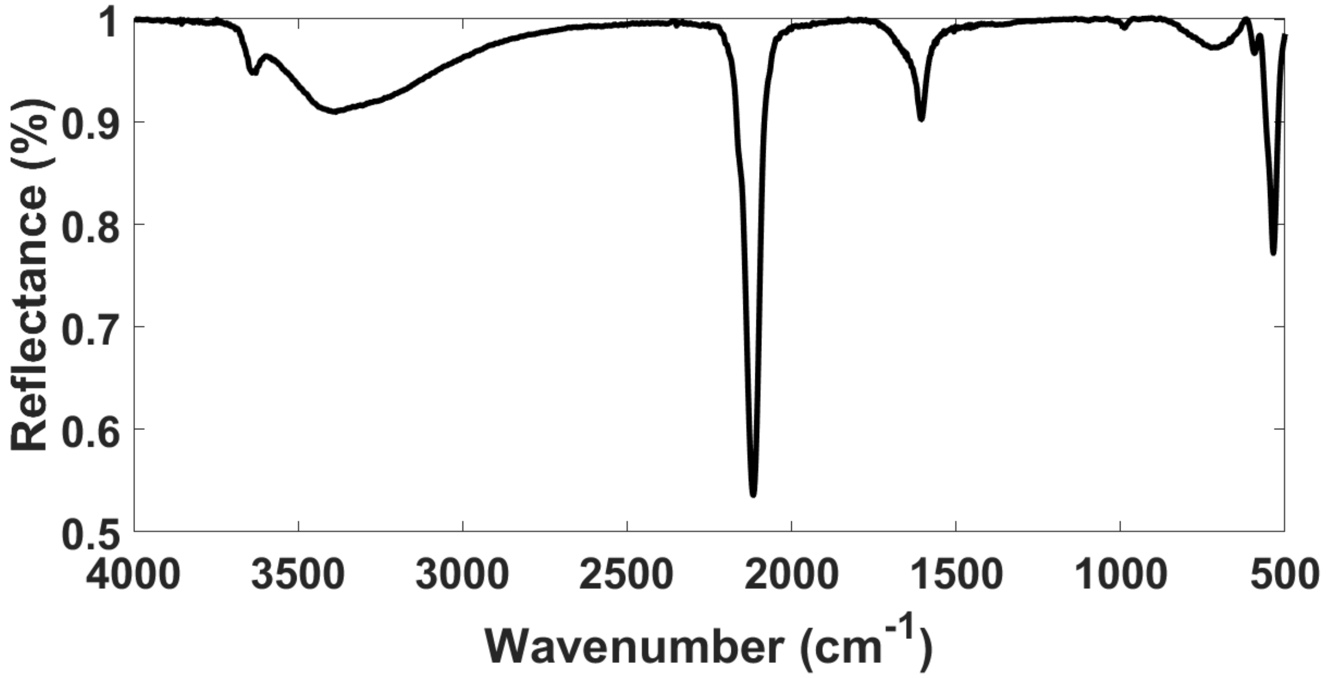

Figure S5. Attenuated total reflectance Infrared spectrum of PBA.

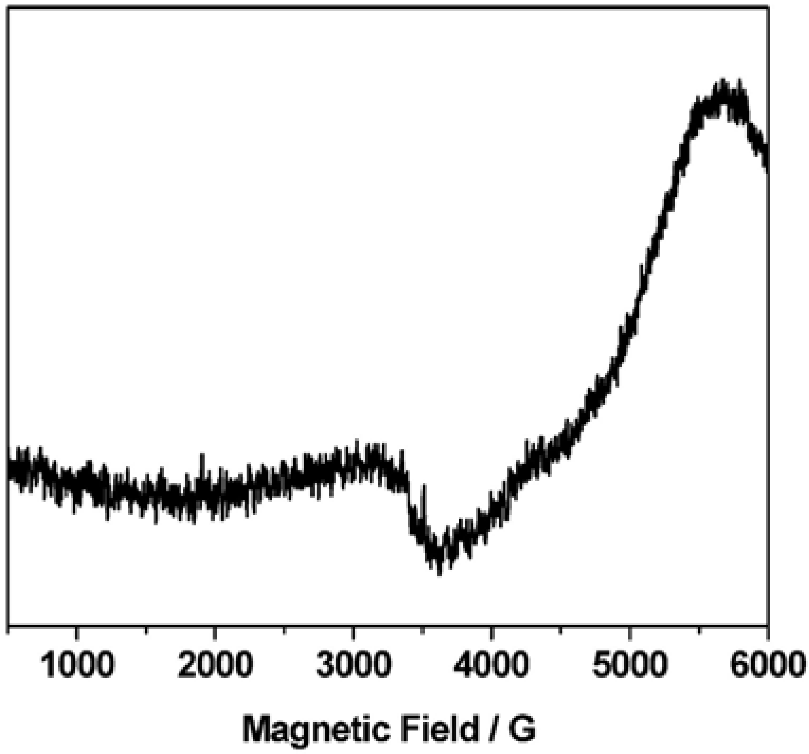

Figure S6. The electron paramagnetic resonance spectrum of PBA at room temperature. The measured signal arises from the cavity of the instrument (no sample signal observed). The lack of clear EPR signal, despite the presence of Fe(III)-LS (S=1/2) and Co(II)-HS (S=3/2) paramagnetic centres is typical of PBA analogues and is likely due to rapid spin-lattice relaxation driven by charge fluctuations persisting above the LT-HT phase transition state.[7]

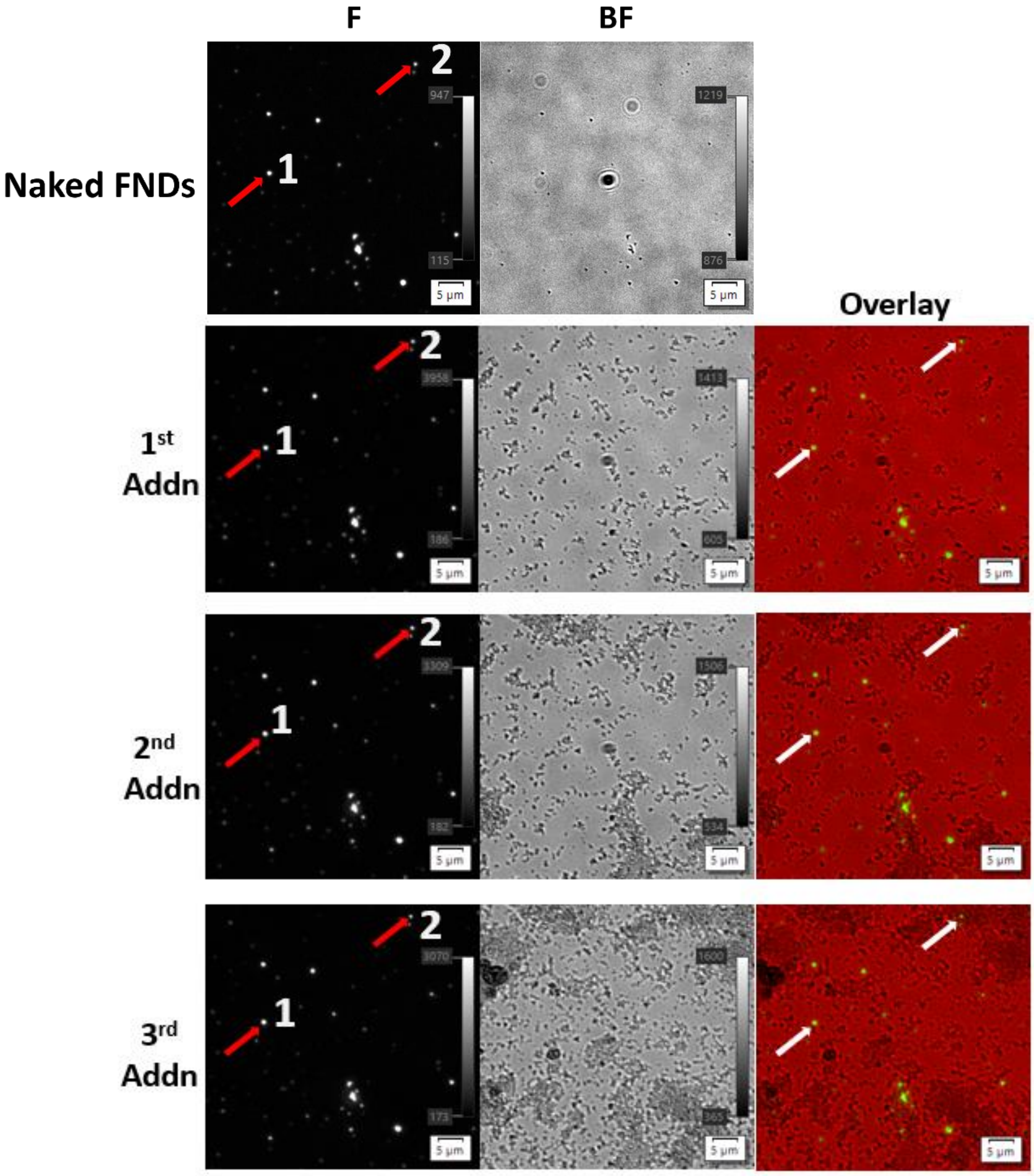

Figure S7. Fluorescence (F), brightfield (BF) and overlay (F+BF) images of drop cast FNDs and rubidium cobalt iron Prussian blue analogue (PBA) nanoparticles. In each successive addition more PBA NPs are deposited onto the field of view. In the 3rd addition, a substantial amount of PBA particles landed near the vicinity of FND cluster 2. No PBA NPs land on or in the vicinity of ND cluster 1 (this gives a diamagnetic ODMR and MM signal – as seen in **Figure S8** – therefore acting as an internal control. FND cluster 2 gives a paramagnetic response which increases after more drop cast addition of the PBA.

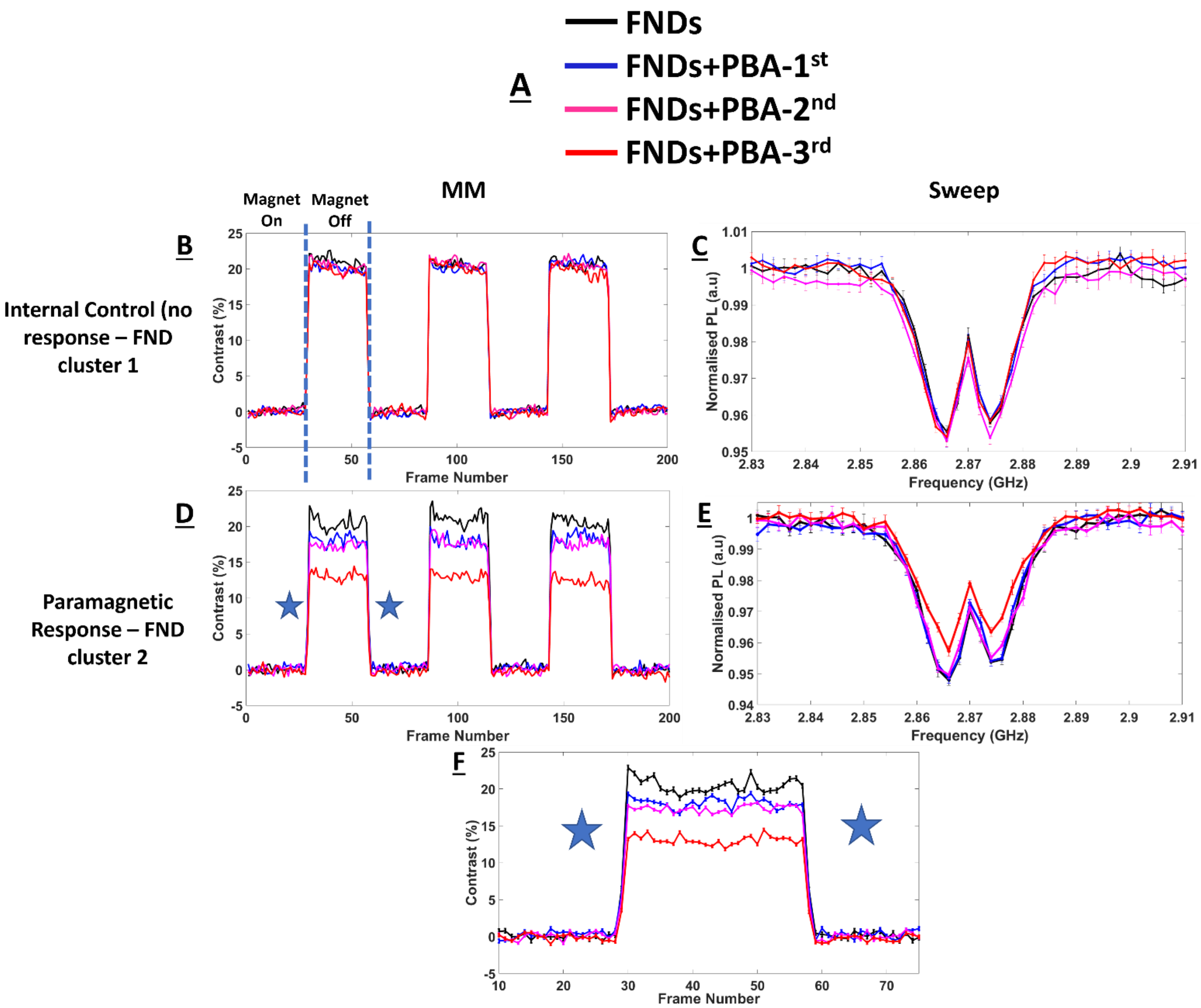

Figure S8. The ODMR (sweep) and MM data for FND cluster 1 (no response) and cluster 2 (observed paramagnetism) highlighted in **Figure S7**. A) Legend showing black, blue, magenta and red lines corresponding to the FNDs, FNDs+PBA first addition, FNDs+PBA second addition and FNDs+PBA third addition respectively of the addition of PBA particles. Optical readout for the internal no response control cluster is shown in B) ODMR and C) MM. Optical readout for the paramagnetic response D) ODMR and E) MM. F) An expanded plot of D). The expanded area is indicated by blue stars.

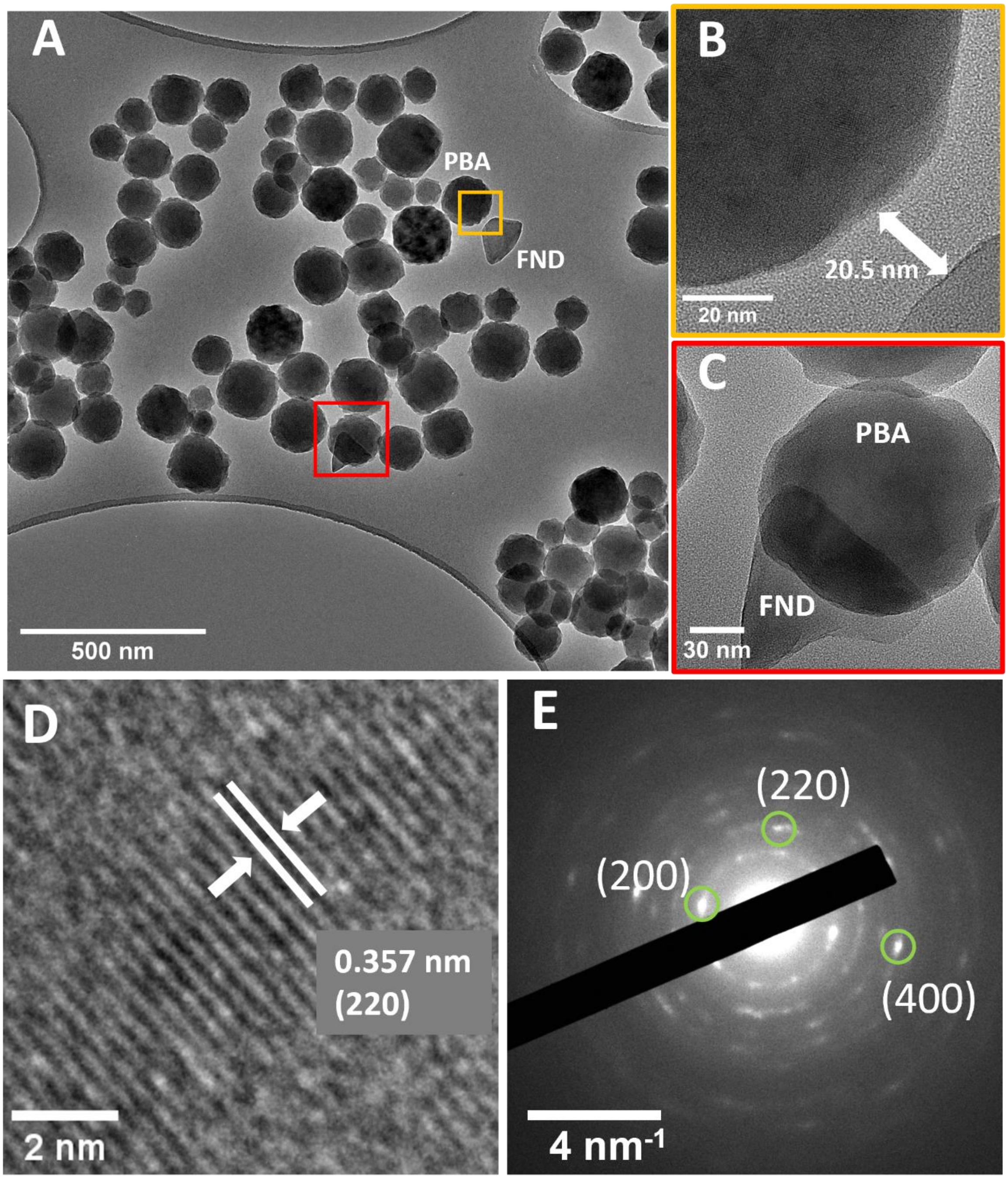

Figure S9. TEM images of PBA NPs and FNDs dispersed onto a Copper lacey carbon finder grid. A) Large field of view image of the dispersed PBA NPs and FNDs achieved by optimised deposition procedure (see experimental section). Two areas on this image have been chosen for further analysis. B) The separation between the PBA particle and the FND particle here is 20.5 nm. This separation is relatively large for NV sensing and is not expected to cause large PL contrast reduction due to paramagnetic interaction using current detection devices. C) Here, the FND particle and the PBA particle appear to form direct contact (or very close proximity). In this case a paramagnetic interaction is expected to cause greater PL contrast reduction. D) High-resolution micrograph of a PBA particle. The (220) atomic plane is identified, marked with arrows and indexed. E) SAED pattern showing diffraction spots corresponding to more atomic planes of the PBA lattice, (200), (220) and (400).

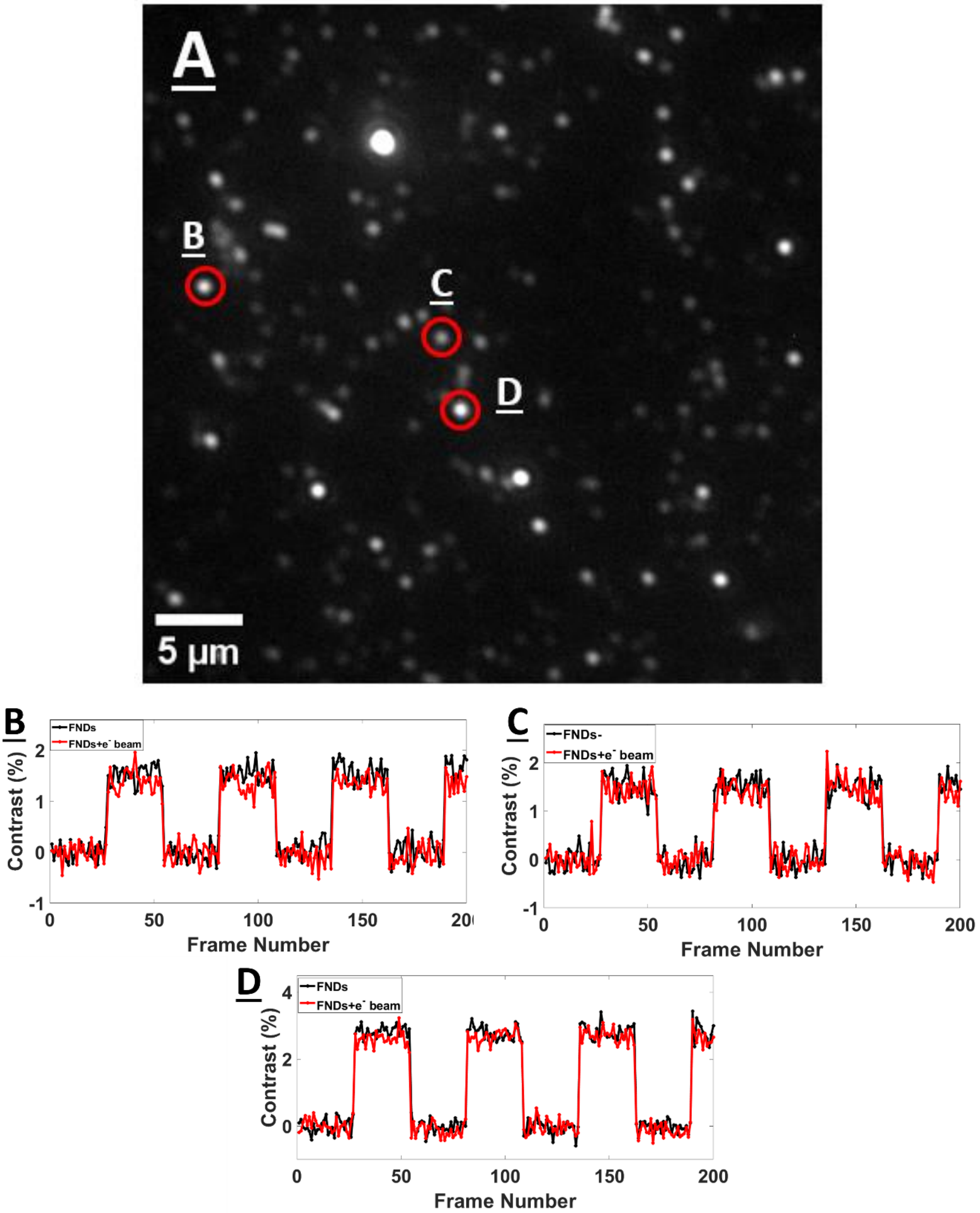

Figure S10. A) A fluorescence image of FNDs drop cast on a TEM grid. Magnetic modulation traces before and after TEM analysis (e^- beam irradiation) has been performed for three FND clusters or single particles (B, C and D). FND clusters here were chosen as they are individual particles or very small FND clusters, plots were not background subtracted (see **Figure S15**) for clarity. No measurable changes were detected after irradiation times of several minutes with low electron flux (50-1000 e- nm^{-2} s^{-1} – see table S2) on the FNDs. However, in the case of high electron flux (>1×10^{6} e- nm^{-2} s-1) and/or long irradiation times (>10 minutes), NV^- centre PL is quenched or turned off. Our results are in agreement with a study by Prabhakar et al. reporting that green emitting FNDs can withstand relatively mild e-beam irradiation conditions.[8] A similar control experiment was carried out on PBA NPs where EDX spectroscopy was used to evaluate e-beam damage, following changes in atomic ratios of Rb, Co, Fe and N at low, medium, and high e-beam flux to monitor the beam damage (SI file, Table S2). The presence of hydrogen atoms in the structure from defect site water molecules can

exacerbate e-beam damage in the PBA lattice, by the same mechanism as shown for H_2O in carbon nanomaterials.[9] The decrease in N content indicates the Fe-C-N-Co linkages are susceptible to e-beam damage and metallic Fe/Co nanoclusters are formed on the PBA particle due to the removal of light elements (O, N) and weakly bound atoms (Rb) by the direct knock-on effect of the e-beam.[10] The final product of high flux e-beam damage on PBA appears to be Co and Fe oxides (an example of this process is shown in Figure S11). Taking on board results of these control experiments, our experiments described in the main text were designed to avoid the e-beam damage in the sample by employing relatively mild e-beam conditions (flux <10,000 e- nm^{-2} s^{-1} and exposure times <5 mins).

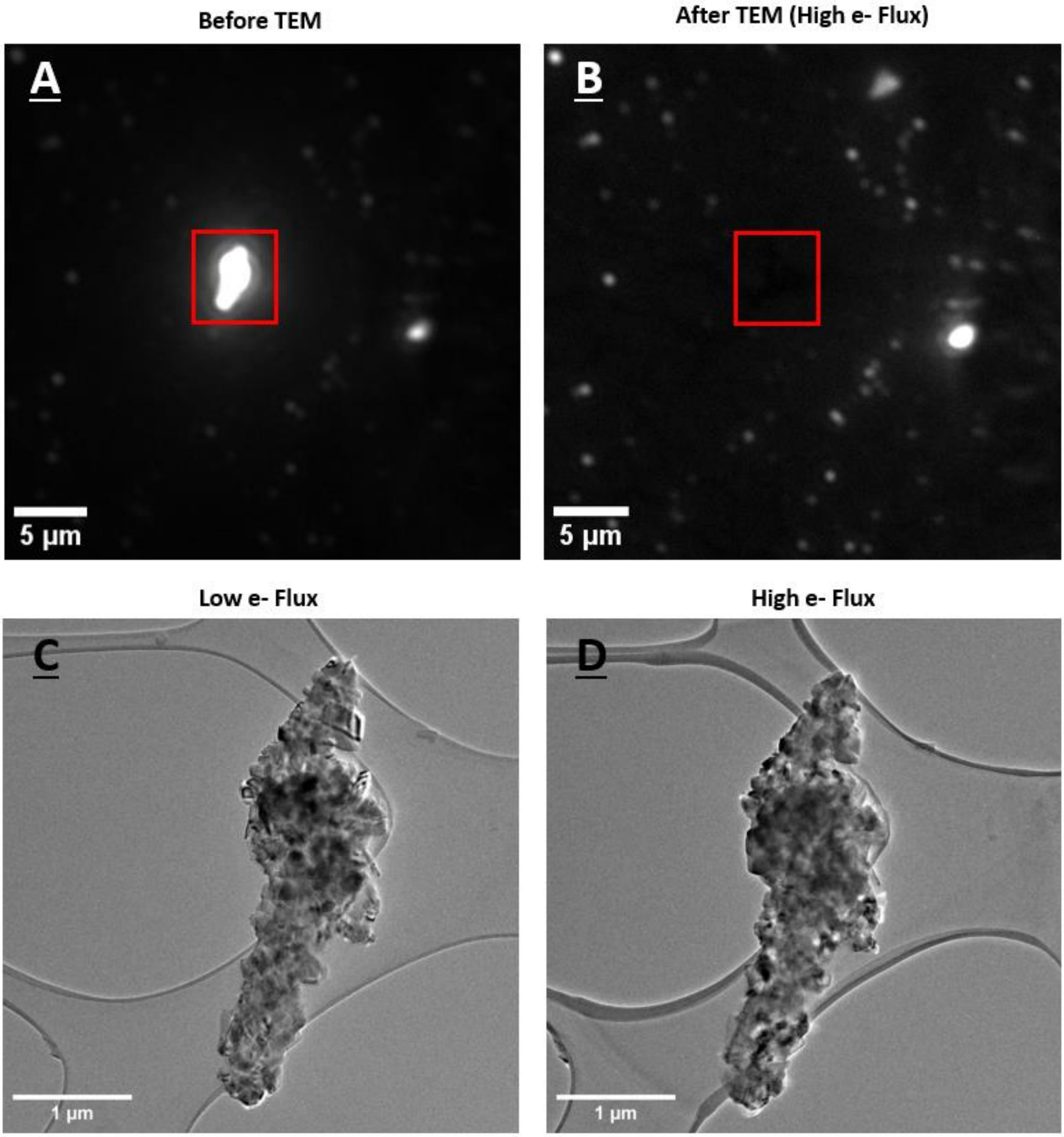

Figure S11. Before TEM (A) and after TEM (B) fluorescence images of a large FND cluster (using high electron flux values close to those explained in table S2 and in the main text, the exposure time in this case was 10 minutes). Low electron flux (C) and high electron flux (D) TEM images of the large FND cluster. When using high electron flux and exposure times (exposure time being how long the electron beam was incident on the region of interest), FND fluorescent properties change (turn off) as described in the main text.

Table S2. Energy dispersive X-ray spectroscopic atomic percentage of elements for PBA particles at different electron flux. Arrows next to element percentages indicate whether the percentage has decreased with increasing electron flux (red) or increased with electron flux (green). Beam damage mechanisms and references can be found in the main text. Beam irradiation time on sample was kept constant for each flux (approximately 1-3 minutes).

Electron Flux (e nm^{-2} s^{-1})	Energy Dispersive X-ray Spectroscopy Atomic Percentage of Elements (%)
Low - 63	N: 52.5<br>O: 21.4<br>Co: 11.9<br>Fe: 9.0<br>Rb: 5.3
Medium - 967587	N: 51.7 ↓<br>O: 19.7 ↓<br>Co: 12.9 ↑<br>Fe: 9.8 ↑<br>Rb: 5.9 ↑
High - 2203875	N: 47.9 ↓<br>O: 21.0 ↑<br>Co: 13.9 ↑<br>Fe: 10.7 ↑<br>Rb: 6.4 ↑

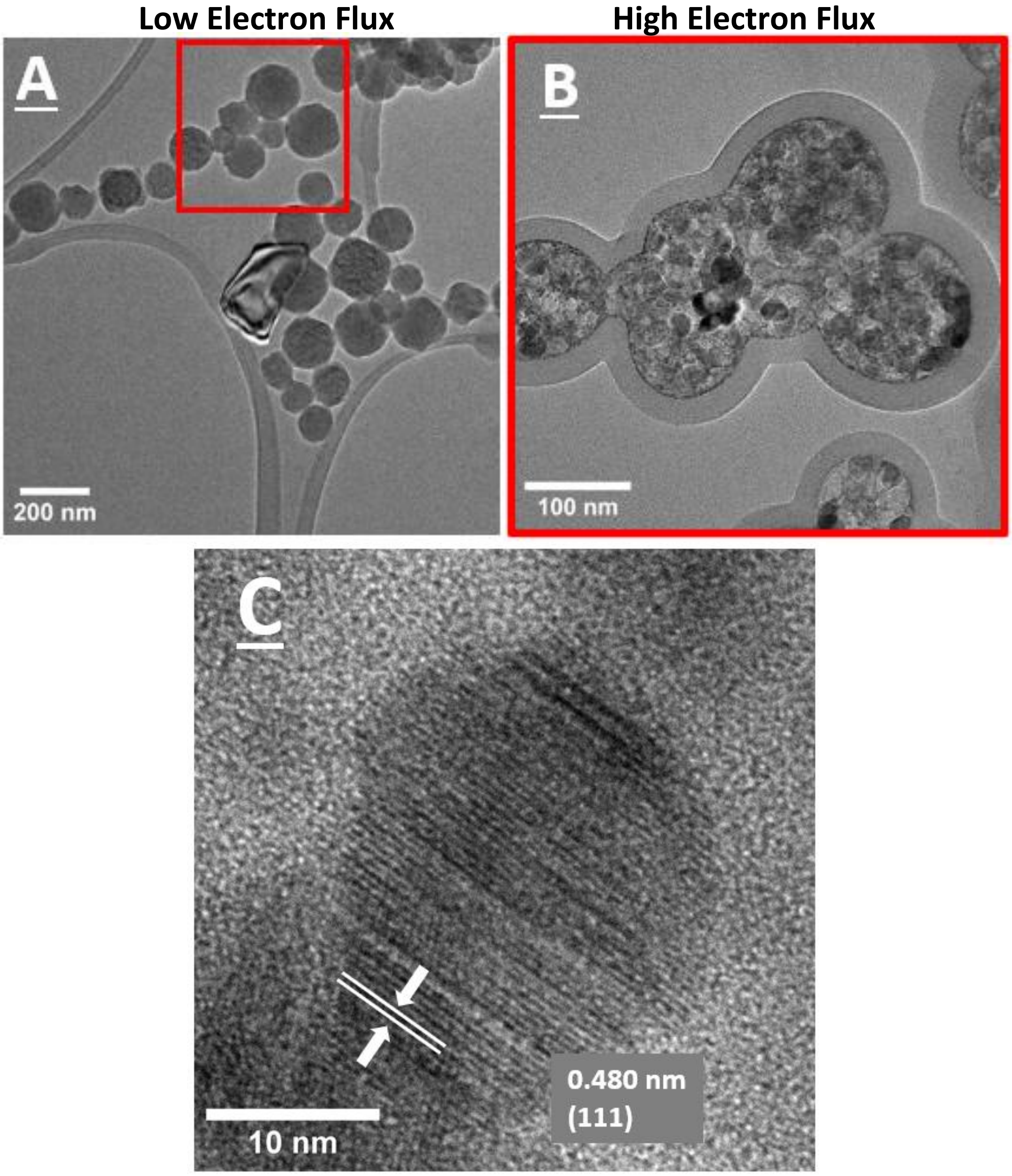

Figure S12. A) Low electron flux TEM image of nanoparticle of **1**. B) High electron flux TEM image showing the effects of beam damage on the same nanoparticles as seen in A). 'Low' and 'high' electron flux values can be seen in table S2. C) An arbitrary zoomed in region of B) showing the product of beam damage on the PBA material, shown here is a magnetite (Fe_3O_4) nanoparticle. d-Spacing for the (111) atomic plane is indexed and marked with arrows. Low/high flux were similar to those in **Table S2** and in the main text.

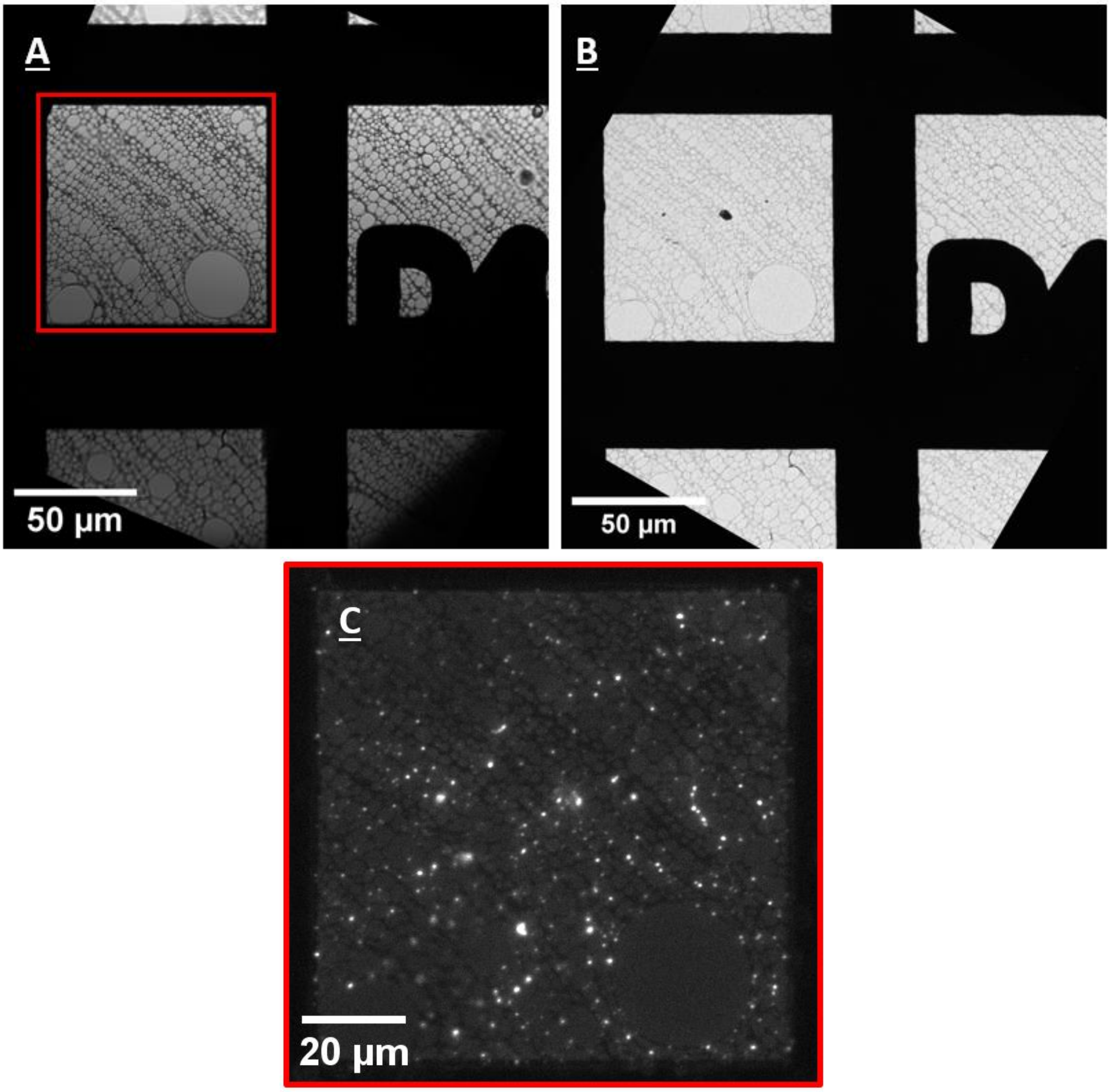

Figure S13. Low magnification optical (A) and electron (B) microscopy images of an example alphanumerical (finder) grid focused on the grid square to the left of 'D4'. Alphanumerical grids can be used to re-locate areas of interest between different iteration of the same technique or between different characterisation techniques. C) is a zoomed in photoluminescence image of the brightfield image A). The FNDs can clearly be seen as bright spots varying in size from single particles to large clusters.

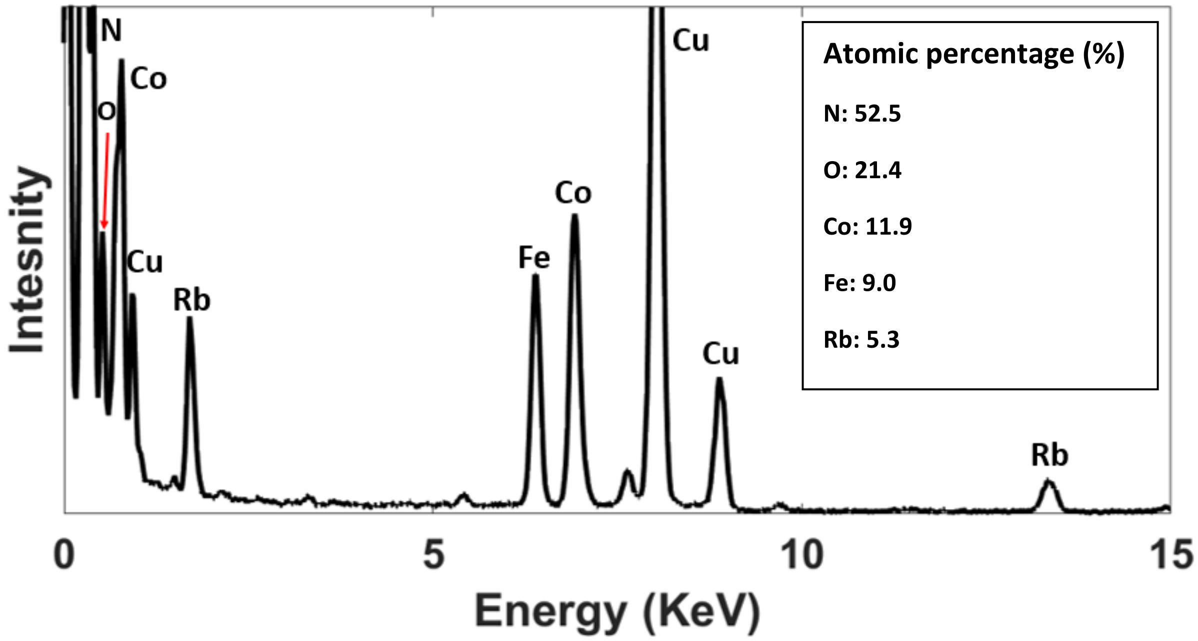

Figure S14. The energy dispersive X-ray spectrum of a PBA particle. Cu signal is from the metallic frame of the TEM grid/in the TEM column. C is present in the film support therefore has been deconvoluted from atomic % calculation.

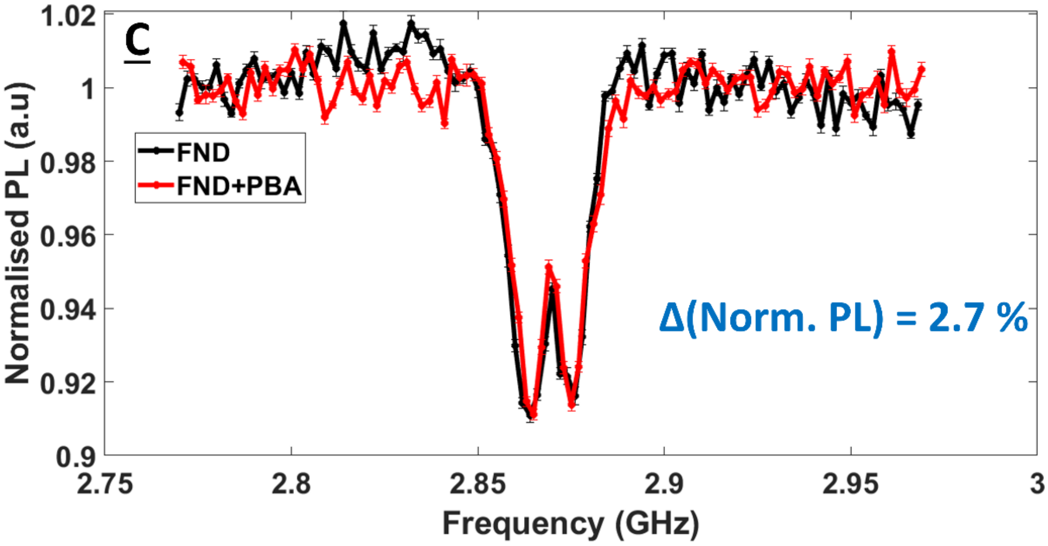

Figure S15. The optically detected magnetic resonance spectrum of the single nanodiamond which is not in contact or close proximity to any spin active PBA material (same FND as shown in figure S18). The signal was background subtracted the same way as **Figure 5** in the main text, which is explained in more detail in **Figure S15**.

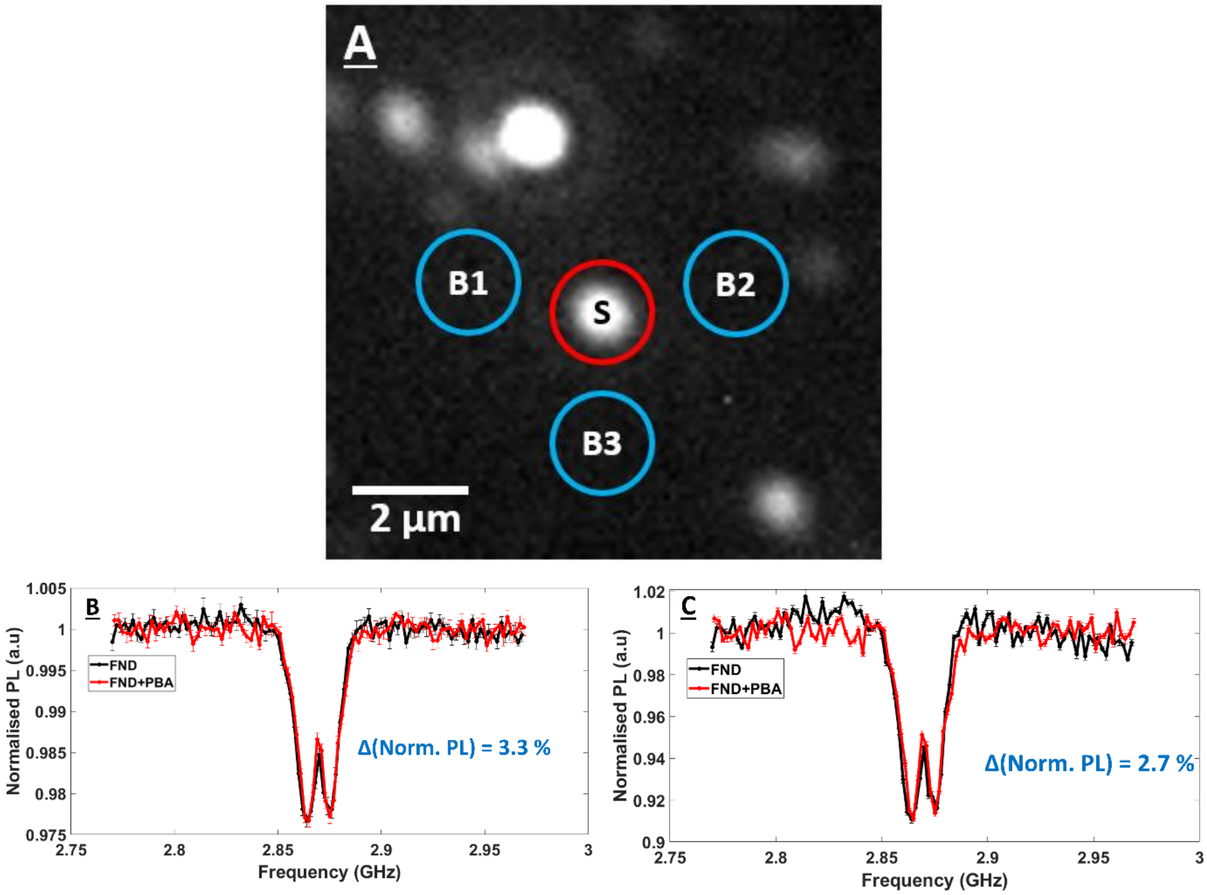

•

Figure S16. An example of how background subtraction is performed on magnetic modulation or optically detected magnetic resonance data. Shown her as a single PL image (A) but in reality, there is a stack of images. Background (B1, B2 and B3) from three areas around the target signal (S1) are averaged and subtracted from S1:

Background Subtracted Data = S – (B1+B2+B3)/3.

An example of an ODMR data set is shown for the single FND that is not in contact with any PBA material. The raw data is shown in B) and the background subtracted data is shown in C). Background subtraction takes into account any difference in background light that is emitted from nearby FND clusters or potential reflections from the metallic grid bar. The effects of background subtraction here are subtle. However, this gives a more accurate representation of the local FND environment being studied. Areas of background are taken at distances beyond the NV sensing range (>100s of nm). With this subtraction, contrast values are artificially enhanced by removing background signal.

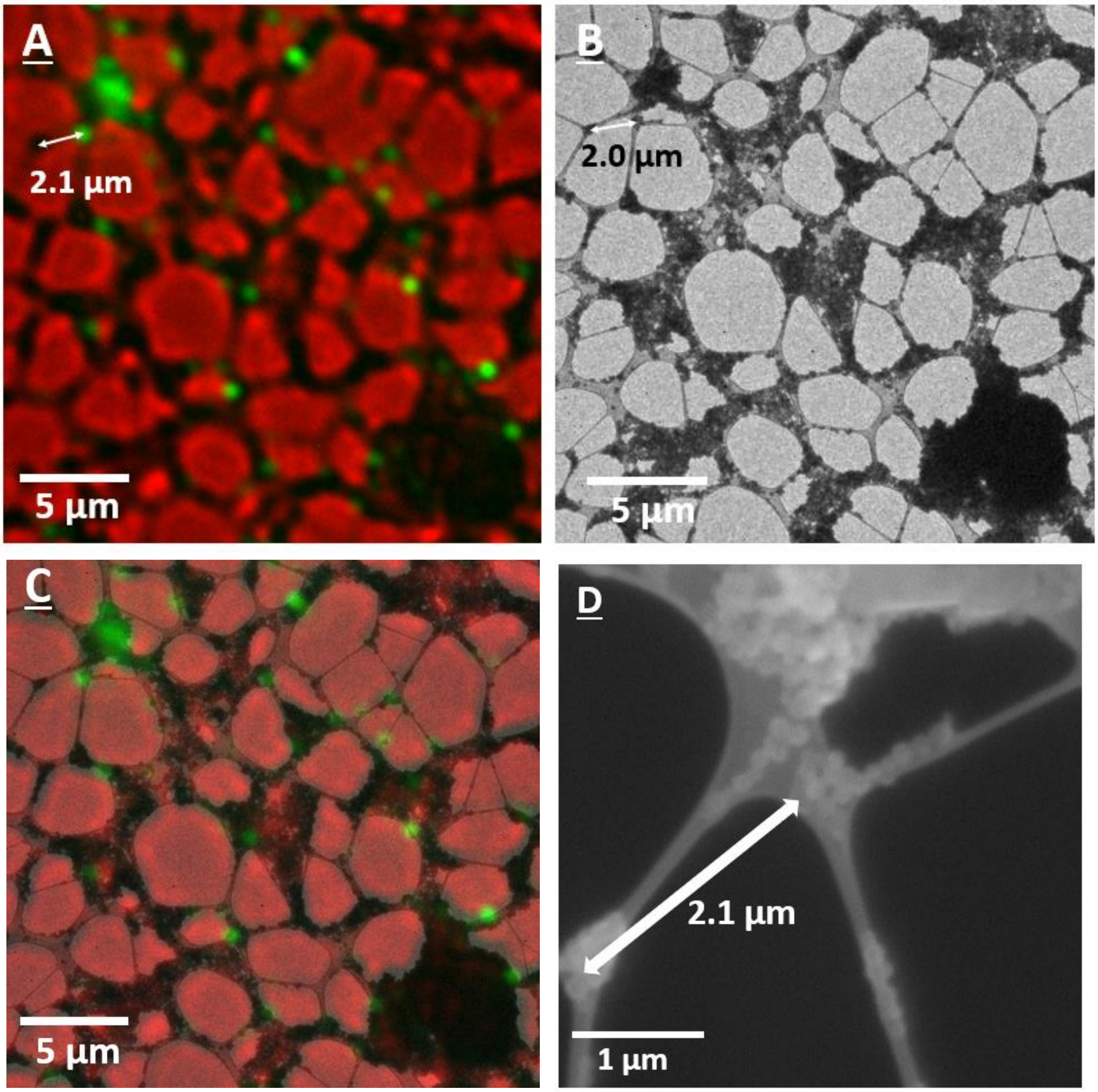

Figure S17. Typical non-integrated correlative light-electron microscopy, confirming PL FND spots as show in A) can be re-located as the single FND imaged in TEM B) and in SEM D) (note, it is believed that surfaces of nanoparticles became coated with an amorphous deposit after SEM measurements, areas away from the field of view did not have this effect – SEM images were taken after NV sensing measurements, so the target ODMR and MM results is not affected).[11] In A) distances are measurements from the centre of a cluster of NPs to the centre of the PL spot (2.1 µm). This is then repeated in low magnification TEM and STEM imaging giving 2.0 µm and 2.1 µm respectively. Using features of the carbon film and features of NPs we are able to re-locate FNDs of interest between microscopy methods. C) Shows an overlay of A) and B), indicating the exact position of the single FND on the carbon film. Similar relocation methodology was repeated throughout this work.

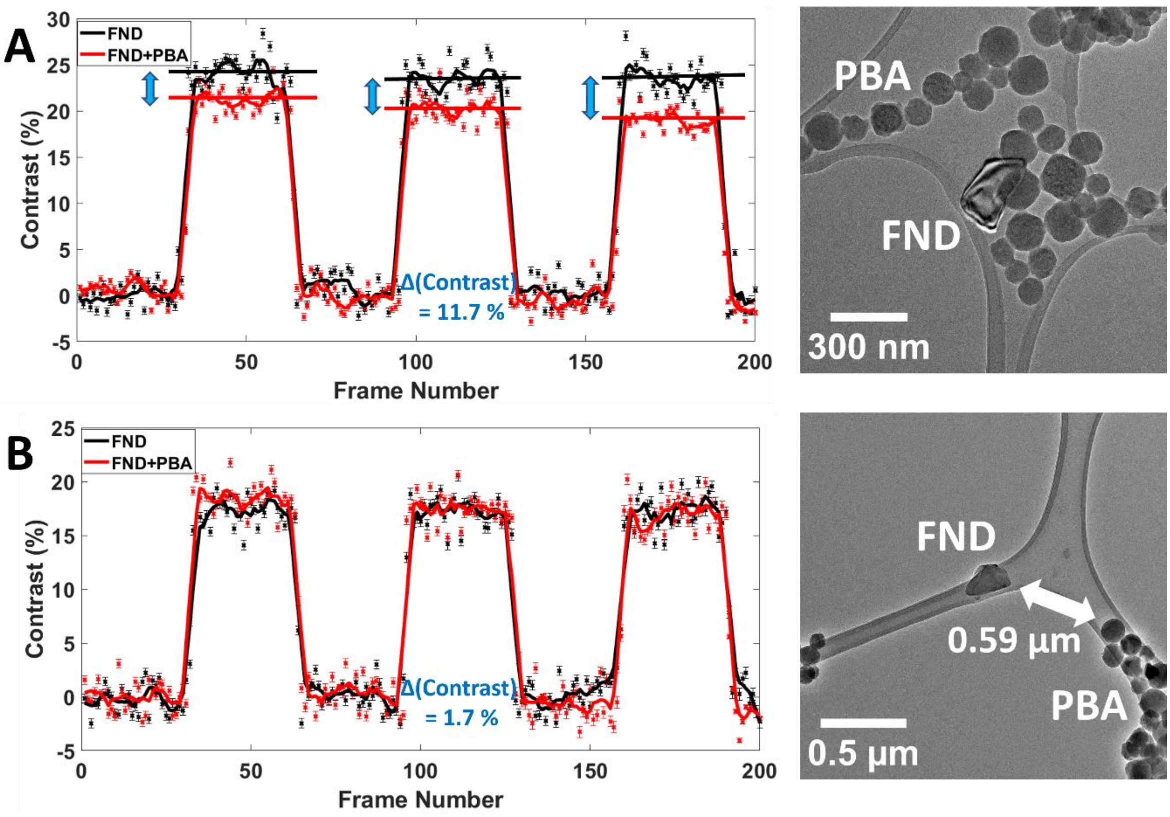

Figure S18. MM traces for a single FND particle that gives a paramagnetic response (A) and no response (B), with corresponding TEM images confirming the presence or absence of spin active PBA NPs respectively. ODMR traces agree with this result (Figure 5I for A and SI, Figure S14 for B). For both MM and ODMR, the difference between the FND and FND+PBA PL signal is shown in blue (%). For MM traces the difference is averaged across the three magnetic cycles.

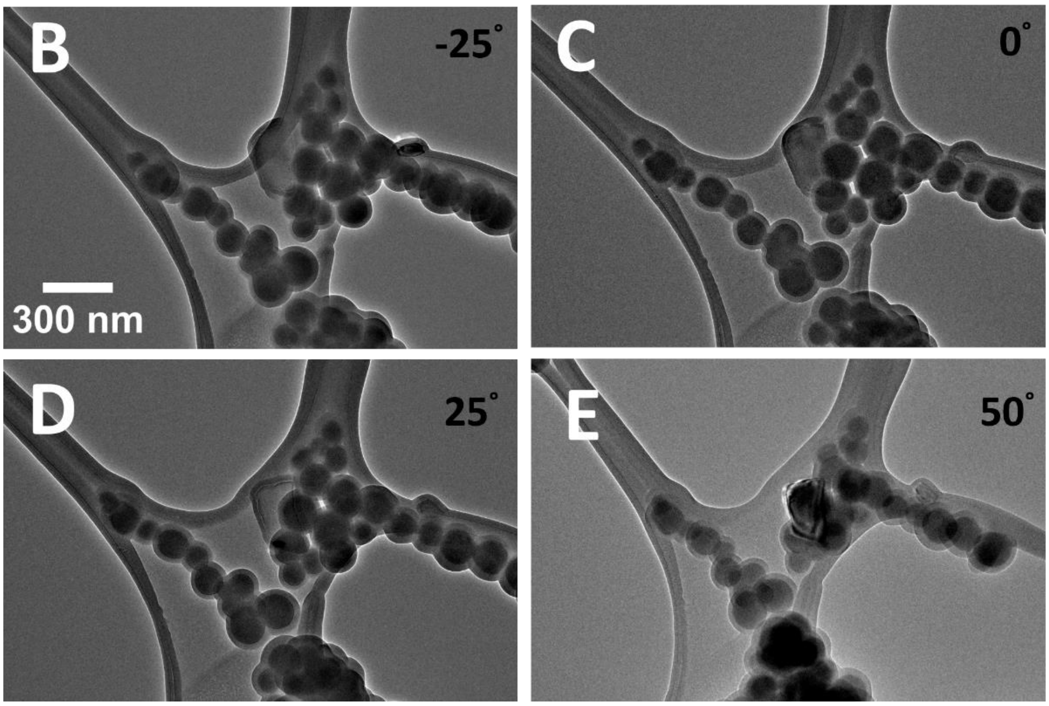

Figure S19. BF-TEM tilt series images (top right, around the x-axis of the stage holder) of the interacting single FND and PBA NPs. The single plate-like FND particle is on the opposite side of the carbon film to the spherical PBA NPs. Videos of tilt series both in BF-TEM and DF-STEM are the SI section. (Note that surfaces of NPs became coated with an amorphous deposit after SEM measurements, taken after NV sensing measurements, details are in SI file, Figure S16).

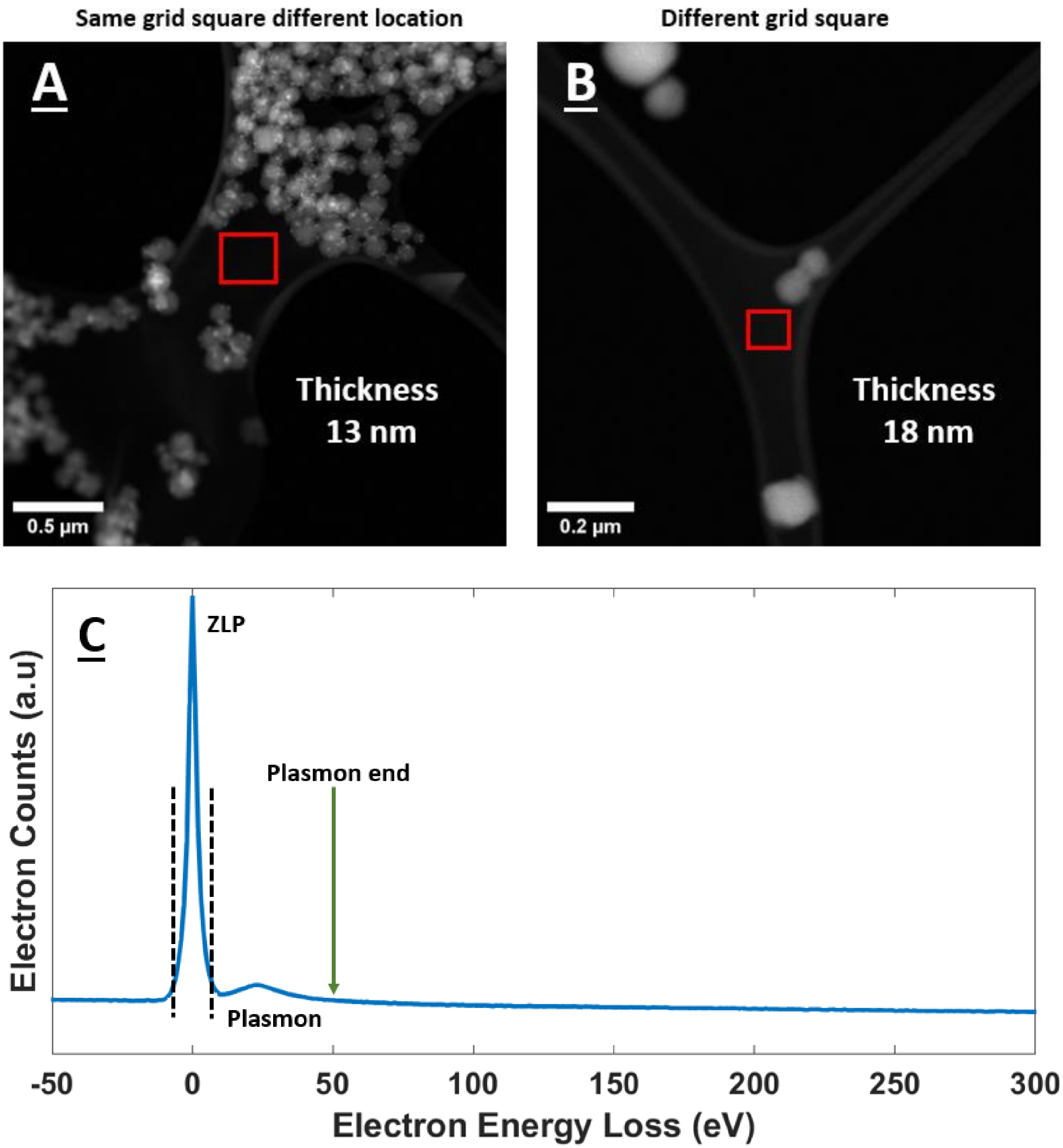

Figure S20. STEM images (A and B) and carbon film thickness values away from the area of interest in the main text, measured using electron energy-loss spectroscopy (EELS). Red boxes indicate the area in which the STEM-EELS was taken. Accurate background subtraction and thickness measurements were performed using an adapted published code[12], which can be found in a separate file in the additional files section. This code does not account for plural electron scattering and therefore is only suitable for thin samples. C) The EELS spectrum from the area in shown in A). The zero-loss peak (ZLP) was integrated between the full width half maxima (FWHM) and where the spectrum meets the baseline, i.e the tail (in this specific example the integration range was 18 eV). Where the plasmon peak meets the baseline was denoted as the plasmon end value.

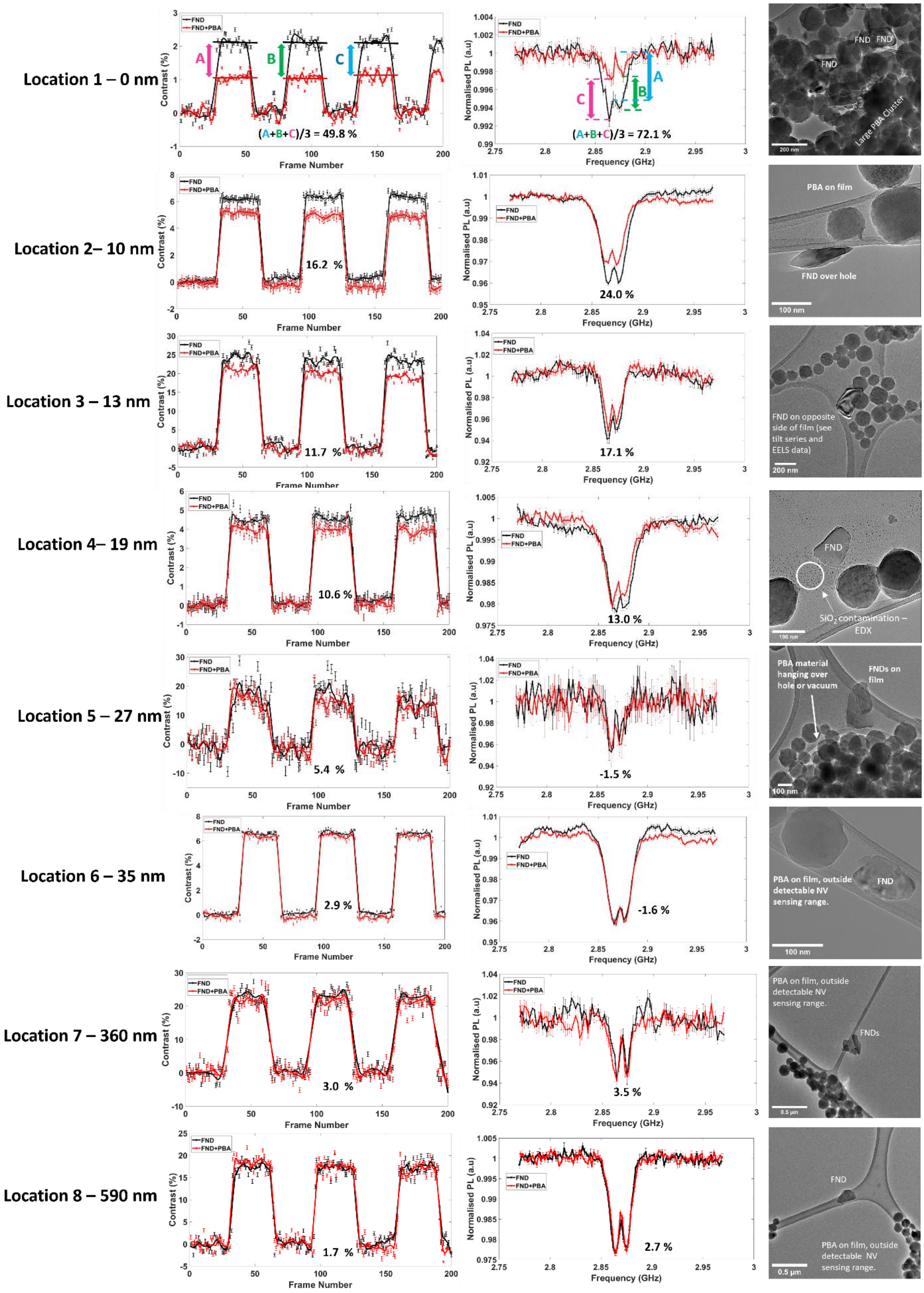

Figure S21. ODMR spectra and MM traces for areas 1-8 with corresponding TEM images. The percentage change in PL before vs after addition of PBA is shown in %. For MM, the difference was

averaged across three magnet ‘off’ cycles. For ODMR the change was quantified by taking averaged data points over the double minima and the resonant frequency values (these specific data points have been averaged over 10 individual points – therefore already have an associated error). For some locations, background subtraction (explained in **Figure S15**) was not applied. For example, it is not clear in location 1 (due to the vast amount of overlapping PBA nanoparticles) precise locations of all FNDs in this region of interest (ROI) measurement, so choosing areas for ‘background’ was not trivial. As addressed in **Figure S15**, background subtraction often only has a minor effect on contrast change (%) when performing before and after addition of PBA analysis. See **Table S3** for all numerical data (FND-PBA separation, change in PL, errors).

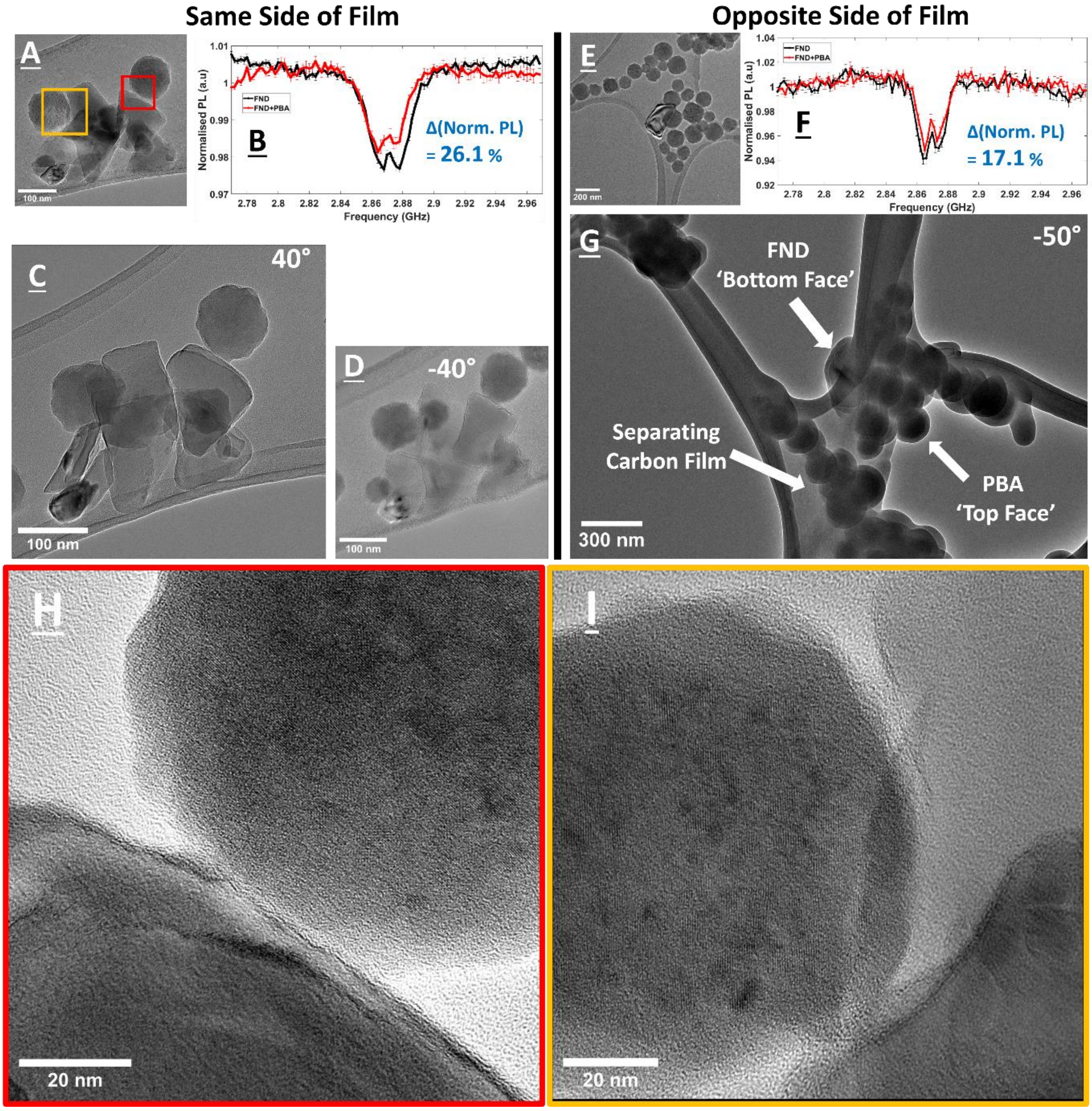

Figure S22. Traditional 2D TEM images of two FND-PBA locations A and E. C and D show TEM tilt series analysis for the area shown in A. This analysis shows FND-PBA particles in direct contact on the same side of the amorphous carbon film. G shows tilt analysis of the area show in E. In this region, as state in the main text, the FND and PBA particles are on different sides of the carbon film. This is important to consider when conducting local NV sensing analysis as shown in the ODMR plots B (change in PL 26.1 %) and F (change in PL 17.1 %), for the areas A and E respectively. From first 2D inspection both locations seem to have FND-PBA particles in direct contact, but their ODMR response is significantly different. Studying this system using TEM tomography allows this difference to be justified. Digitally rotating tilt series can be found in the additional files, this especially helps for location A, some FND-PBA particles rotate together paradoxically (same plane) unlike location E. H and I show Zoomed-in areas of A (these points can be followed in the rotating video file to show they move together, i.e in direct physical contact, interparticle separation assumed 0 nm).

Table S3. A table of extracted numerical values of contrast change for both NV sensing schemes, MM and ODMR, as a function of FND-PBA separation for 8 locations imaged in the main text.

**Location (with separation in nm)**	**NV Sensing Technique**	
	**MM**, change in FND PL upon PBA addition (%)	**ODMR**, change in FND PL upon PBA addition (%)
1 (0)	50 $\pm$ 3	72 $\pm$ 6
2 (10 $\pm$ 5)	16 $\pm$ 2	24 $\pm$ 3
3 (13 $\pm$ 5)	12 $\pm$ 2	17 $\pm$ 2
4 (19 $\pm$ 4)	11 $\pm$ 2	13 $\pm$ 2
5 (27 $\pm$ 3)	5 $\pm$ 1	-2 $\pm$ 2
6 (35 $\pm$ 3)	3 $\pm$ 1	-2 $\pm$ 2
7 (360 $\pm$ 5)	3 $\pm$ 1	4 $\pm$ 2
8 (590 $\pm$ 10)	2 $\pm$ 1	3 $\pm$ 1

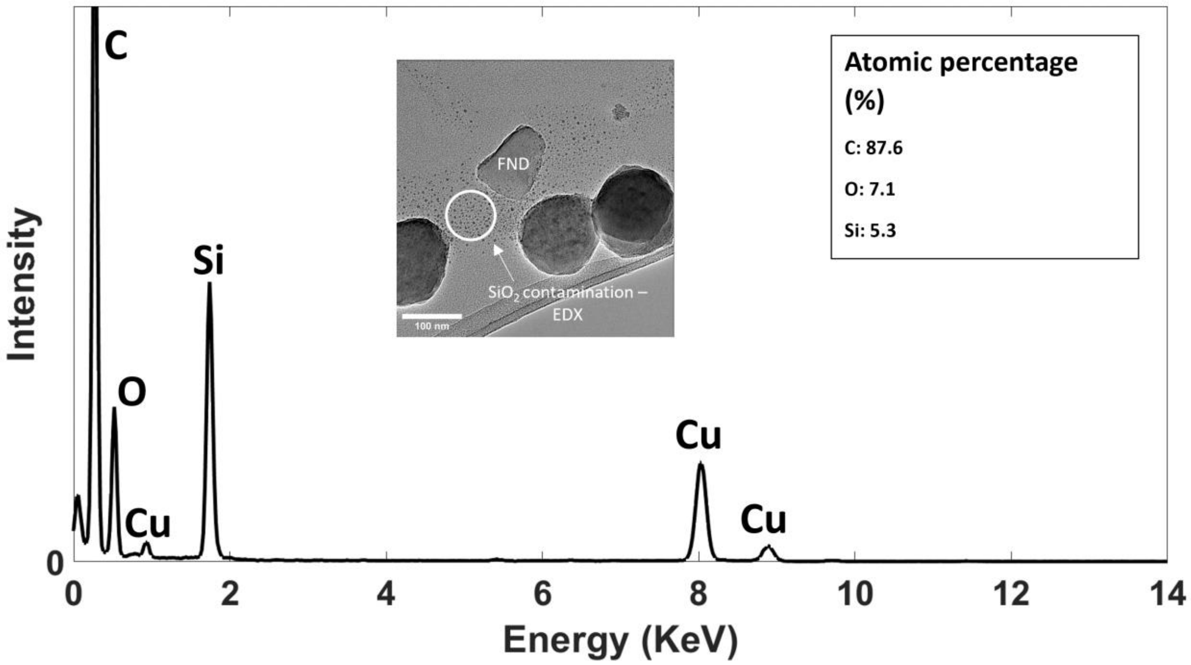

Figure S23. The energy dispersive X-ray spectrum of the area located in the TEM images (white circle). Silica is a common contaminant found in carbon support on TEM grids, either through laboratory contamination or fundamentally from the TEM grid fabrication process. Silica is well known to be diamagnetic and therefore is not likely to affect ODMR or MM paramagnetic sensing measurements. Cu signal is from the metallic frame of the TEM grid which is deconvoluted from atomic percentage calculations.

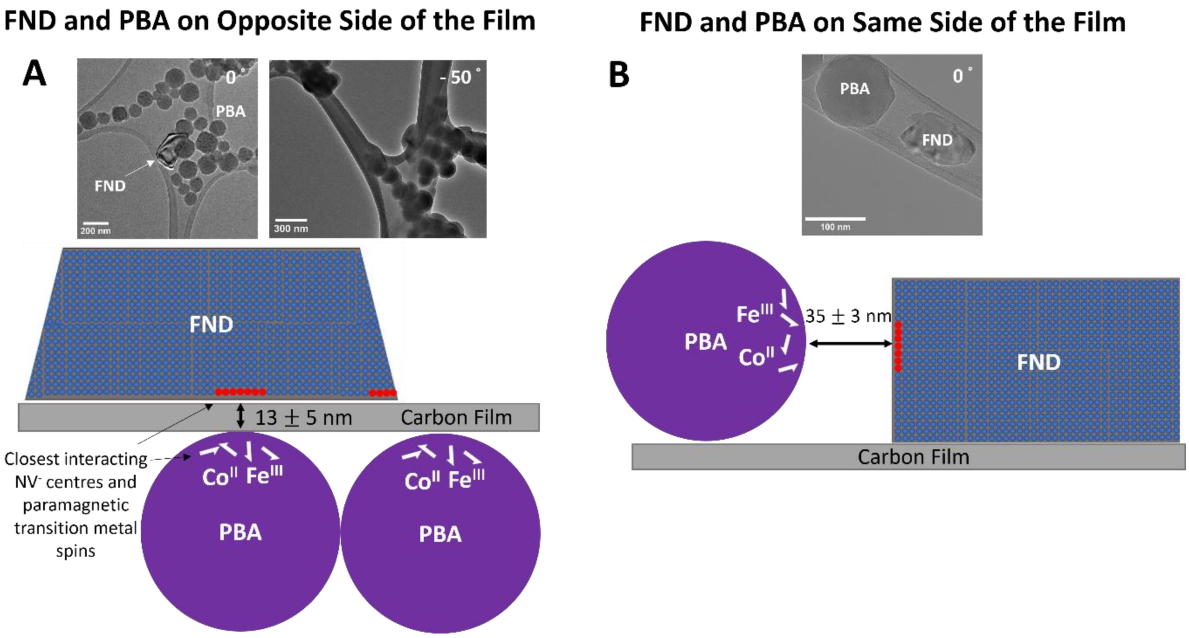

Figure S24. Schematic explanation of the two different distance measurement protocols, with errors, used in **Figure 6** of the main text. Diagrams (not to scale) are viewed side on to 0 ° tilt TEM images to illustrate the importance of opposing side film location when using local NV sensing. PBA particles are represented as spheres (circles in 2D) and FND particles used here roughly represent the 2D shape of the particle as seen in the corresponding TEM image. FND particle shape can be irregular.[13] Blue circles within FND particles represent the assumed even distributed of NV centres ($\approx$ 1000 – as quoted by supplier, FND Biotech, see main text). Scenario A (location 3 of **Figure 6**) has FND and PBA particles that appear to be in direct contact viewed down the axis of the beam. Tilt series analysis (-50 °, TEM tilt in the x-axis of the holder is shown in the top right of the micrographs) confirms FND and PBA particles to be on opposite sides of the film. Particles lie flat on the film, so the shortest edge-to-edge FND-PBA separation, containing groups of spins – PBA Co and Fe paramagnetic spins (shown by randomly orientated white half electron arrows) and FND NV^- centres (indicated by red circles) - on the surface, is defined by the thickness of the carbon film, measured by EELS to be 13 $\pm$ 5 nm ($\bar{x} \pm 2\sigma$). Scenario B shows location 6 of Figure 6 in the main text, where FND and PBA particles are on the same side of the carbon film, therefore closest FND-PBA edge-to-edge interparticle separation can be measured directly from 2D TEM micrographs. As this distance measurement is from shortest edge-to-edge separation, irregularity and distribution of size in FND and PBA particles becomes significantly less important. Distance error: 3 edge-to-edge distance line profiles between particles were drawn and averaged, the total uncertainty in distance measurements comes error propagation through the ability to measure pixel-to-pixel edges in 2D micrographs and standard deviation errors of the 3 averaged distances.

References

1. H. J. Maeng, D. H. Kim, N. W. Kim, H. Ruh, D. K. Lee and H. Yu, *Current Applied Physics*, 2018, **18**, S21-S27.
2. T. G. F. Souza, V. S. T. Ciminelli and N. D. S. Mohallem, Bento Goncalvesalves, BRAZIL, 2015.
3. Y. F. Xia, G. Zhang, M. Liu and D. X. Li, *Journal of Superconductivity and Novel Magnetism*, 2019, **32**, 3831-3835.
4. M. Zentkova and M. Mihalik, *Crystals*, 2019, **9**.
5. A. Bleuzen, C. Lomenech, V. Escax, F. Villain, F. Varret, C. C. D. Moulin and M. Verdaguer, *Journal of the American Chemical Society*, 2000, **122**, 6648-6652.

6. N. Shimamoto, S. Ohkoshi, O. Sato and K. Hashimoto, *Inorganic Chemistry*, 2002, **41**, 678-684.
7. A. Antal, A. Janossy, L. Forro, E. J. M. Vertelman, P. J. van Koningsbruggen and P. H. M. van Loosdrecht, *Physical Review B*, 2010, **82**.
8. N. Prabhakar, M. Peurla, O. Shenderova and J. M. Rosenholm, *Molecules*, 2020, **25**.
9. J. Biskupek, S. T. Skowron, C. T. Stoppiello, G. A. Rance, S. Alom, K. L. Y. Fung, R. J. Whitby, M. H. Levitt, Q. M. Ramasse, U. Kaiser, E. Besley and A. N. Khlobystov, *Acs Nano*, 2020, **14**, 11178-11189.
10. R. F. Egerton, *Micron*, 2019, **119**, 72-87.
11. M. T. Postek, *Scanning*, 1996, **18**, 269-274.
12. K. L. Y. Fung, M. W. Fay, S. M. Collins, D. M. Kepaptsoglou, S. T. Skowron, Q. M. Ramasse and A. N. Khlobystov, *Ultramicroscopy*, 2020, **217**.
13. S. Eldemrdash, G. Thalassinos, A. Alzahrani, Q. Sun, E. Walsh, E. Grant, H. Abe, T. Greaves, T. Oshima, P. Cigler, P. Matejicek, D. Simpson, A. Greentree, G. Bryant, B. Gibson and P. Reineck, *ELSEVIER*, 2023, **206**, 268-276.
